# Conditional Gene Targeting Reveals Cell Type-Specific Roles of the Lysosomal Protease Cathepsin L in Mammary Tumor Progression

**DOI:** 10.3390/cancers12082004

**Published:** 2020-07-22

**Authors:** María Alejandra Parigiani, Anett Ketscher, Sylvia Timme, Peter Bronsert, Manuel Schlimpert, Bernd Kammerer, Arnaud Jacquel, Paul Chaintreuil, Thomas Reinheckel

**Affiliations:** 1 Institute of Molecular Medicine and Cell Research, Faculty of Medicine, Albert-Ludwigs-University of Freiburg, Stefan Meier Str. 17, 79104 Freiburg, Germany; maria.parigiani@mol-med.uni-freiburg.de (M.A.P.); anettketscher@gmx.de (A.K.); 2 Faculty of Biology, Albert-Ludwigs-University of Freiburg, Schaenzle Str. 1, 79104 Freiburg, Germany; manuel.schlimpert@sgbm.uni-freiburg.de; 3 Institute for Surgical Pathology, Medical Center-University of Freiburg, Breisacher Str. 115A, 79106 Freiburg, Germany; sylvia.timme@uniklinik-freiburg.de (S.T.); peter.bronsert@uniklinik-freiburg.de (P.B.); 4 Tumorbank Comprehensive Cancer Center Freiburg, Medical Center–University of Freiburg, 79106 Freiburg, Germany; 5 Faculty of Medicine, Albert-Ludwigs-University of Freiburg, Breisacher Str. 153, 79110 Freiburg, Germany; 6 Center for Biological Systems Analysis (ZBSA), University of Freiburg, 79104 Freiburg, Germany; bernd.kammerer@zbsa.uni-freiburg.de; 7 BIOSS Centre for Biological Signalling Studies, University of Freiburg, Schaenzle Str. 18, 79104 Freiburg, Germany; 8 Université Côte d’Azur, C3M Inserm U1065, 06204 Nice, France; Arnaud.Jacquel@unice.fr (A.J.); Paul.Chaintreuil@unice.fr (P.C.); 9 INSERM U1065, C3M, Team: Myeloid Malignancies and Multiple Myeloma, 06204 Nice, France; 10 Equipe Labellisée par la Fondation ARC, 94803 Villejuif, France; 11 Faculty German Cancer Consortium (DKTK), Partner Site Freiburg, Germany and German Cancer Research Center (DKFZ), 69120 Heidelberg, Germany

**Keywords:** breast cancer, lysosome, proteolysis, genetically engineered mice

## Abstract

*Background:* Cathepsin L (Ctsl) is a cysteine protease mainly located within the endosomal/lysosomal cell compartment. High expression of Ctsl indicates poor prognosis in human breast cancer. However, the cell type-specific Ctsl functions responsible for this association remain elusive. *Methods:* Because constitutive *Ctsl^−/−^* mice develop a complex phenotype, we developed a conditional model allowing for cell type-specific inactivation of Ctsl in mammary epithelium or myeloid cells in the transgenic mouse mammary tumor virus (MMTV)-polyoma middle T (PyMT) breast cancer model. *Results:* Ctsl ablation in mammary epithelial cells resulted in delayed initiation and end-stage of cancers. The latter displayed large dead cell areas. Inducible in vitro deletion of Ctsl in MMTV-PyMT-derived breast cancer cells revealed expansion of the acidic cell compartment, alteration of intracellular amino acid levels, and impaired mTOR signaling. In consequence, Ctsl-deficient cells exhibited slow growth rates and high apoptosis susceptibility. In contrast to Ctsl-deficient mammary epithelium, selective knockout of Ctsl in myeloid cells had no effects on primary tumors, but promoted lung metastasis formation. *Conclusions:* Our cell type-specific in vivo analysis provides strong evidence for a cancer cell-intrinsic, tumor-promoting role of Ctsl in primary breast cancer, whereas metastasis is negatively regulated by Ctsl expressed by bone marrow-derived cells.

## 1. Introduction

Proteolysis is a fundamental event at every single stage of tumorigenesis [1,2]. Cysteine cathepsins modulate physiological as well as pathological processes as important components of the intracellular proteolytic network [3]. This protease family is often overexpressed in tumor cells, resulting in their secretion from the acidic cell compartment into the extracellular space [4]. Along with both tumor-promoting and suppressing roles and different cells of origin, cancer model experiments aided the identification of distinct, non-redundant roles of single cysteine proteases in tumor progression. Profiling of single cysteine cathepsins demonstrated their increased activity during defined stages of tumor progression [5]. Some of them, such as cathepsin Z (Ctsz), were found to be upregulated in tumor-associated macrophages (TAMs) following cathepsin B (Ctsb) knockout [6,7]. Cysteine cathepsins released in the tumor microenvironment (TME) promote tumorigenesis in several ways, e.g., by processing different growth factors, cytokines, and chemokines; by cleaving cell-cell junction proteins; or by remodeling the extracellular matrix (ECM) [8,9]. They also play a role in many other tumor-suppressing processes, such as cell death and autophagy, which can contribute to worsening by the development of drug resistance [10]. 

Although the function of cathepsin L (Ctsl) in the complex process of tumorigenesis is not yet fully understood, the upregulation of its mRNA and protein levels especially in breast cancer correlates with a higher risk of relapse, poor therapy outcome, and worse overall survival [11,12,13]. Ctsl has been shown to have many unique cell type-specific functions crucial for the maintenance of tissue homeostasis, which cannot be compensated by other cathepsins or other cysteine proteases. By means of *Ctsl* null mice, the roles of this protease in epidermal homeostasis, hair follicle morphogenesis and cycling, cardiac function, and MHC-II-mediated antigen presentation of cortical thymic epithelial cells were described previously [14,15,16]. Many other substrates of Ctsl lysosomal activity are arising, together with crucial functions in the development and homeostasis of diverse tissues, e.g., as part of vesicles, Ctsl takes part in the proteolytic processing of neurotransmitters and hormones [17,18,19].

Due to its tissue-specific functions, the role of Ctsl in several cancer types is versatile. Tumor promoting effects were reported for the RIP1-Tag 2 pancreatic islet cell carcinogenesis model [20]. This report established a reduction in tumor growth in *Ctsl^−/−^* animals, resulting from the combination of impaired proliferation and enhanced cell death. A further *Ctsl* knockout study using the MycER^TAM^-Bcl_xL_ pancreatic neuroendocrine cancer revealed an impairment in tumor progression toward the latest stages, an increase in tumor cell death, and elevated expression of autophagy markers, together with defective fusion of autophagosomes and lysosomes [21]. In contrast, several other studies revealed protective functions of Ctsl expression towards carcinogenesis. In a report of intestinal tumorigenesis using the Apc^Min^ model, Ctsl deficiency resulted in an increased tumor incidence as a result of the interplay between Ctsl and the tight-junction protein claudin 1 [22]. Consistently, *Ctsl* knockout in two squamous cell carcinoma models showed an earlier onset of tumors accompanied by an increase in tumor burden and invasiveness, which was explained by hyper-responsiveness to growth factor signals and hyper-activation of the MAPK/AKT pathways [23,24]. A previous study using the mouse mammary tumor virus (MMTV)-polyoma middle T (PyMT) breast cancer model revealed a massively enhanced metastatic burden in the lungs following transgenic overexpression of human Ctsl [25].

Multiple approaches have been employed to surpass the complex phenotype caused by the lack of Ctsl in mice and, at the same time, to enable the study of the cell type-specific contribution of Ctsl to carcinogenesis. In order to analyze to what extent Ctsl supplied by TAMs contributes to tumorigenesis, bone marrow from *Ctsl^−/−^* donor mice was transplanted to RIP1-Tag 2 recipient mice. It could be established that the tumor-promoting functions of Ctsl must be derived from either cancer cells or cells other than from the bone marrow [26]. Additional studies highlighted that restoring the Ctsl catalytic activity in epidermal keratinocytes in a tissue-specific manner can counteract the enhanced malignant phenotype observed in *Ctsl^−/−^* skin cancers [23].

Contrary to the aforementioned efforts, the present study reports a direct approach for exploring cell type-specific Ctsl functions in primary cancers by targeting the protease using a Cre/*loxP* strategy. Conditional Ctsl deletion in mammary epithelium and the cancer cells derived therefrom or, alternatively, in myeloid cells capable of infiltrating breast tumors displayed distinct, tissue-specific functions of Ctsl in the maintenance of cell homeostasis, survival, and proliferation in breast cancer. We further provide evidence for an important intracellular function of Ctsl related to lysosomal homeostasis and lysosome-dependent mTOR signaling.

## 2. Results

### 2.1. Generation and Characterization of Conditional Ctsl Knockout Mice

We made use of the Cre/*loxP* technology to address cell-specific functions of Ctsl in murine breast cancer. *Ctsl* was targeted by flanking exons 3–6 with *loxP* sites (Figure S1A III). Cre-mediated recombination was predicted to result in the deletion of those exons and in a frameshift-mutation terminating Ctsl translation (Figure S1A IV).

As a proof of concept, *Ctsl^fl/fl^* mice were crossed with Sox2-Cre mice, thereby giving rise to litters bearing a ubiquitous deletion of Ctsl (Figure S1B). Accordingly, Ctsl protein was absent in the kidney and liver of those animals. Furthermore, Ctsl mRNA levels were also undetectable by primers located between the exons 1 and 4, confirming the accuracy of the Cre/*loxP* strategy for our purposes. We also found the well-described skin phenotype of constitutive *Ctsl* null mice in the Sox2-Cre/*Ctsl^−/−^* animals (Figure S1C). This phenotype is characterized by disturbed hair follicle cycling as well as epidermal thickening due to keratinocyte hyperproliferation and hyperkeratosis, thereby contrasting with normal skin in the *Ctsl^fl/fl^* mice [15].

In the next step, we deleted Ctsl specifically in mammary epithelium and consequently in the cancer cells originating from them, or, alternatively, in myeloid cells. This was achieved by tissue-specific expression of the Cre recombinase directed either by the MMTV-long terminal repeat or the myeloid cell-specific lysozyme M (LysM) promoter, respectively [27,28]. The use of these cell-specific Ctsl deletions enabled us to overcome the defective positive selection of T helper cells at the thymic epithelium of *Ctsl* null mice [14]. This was proven by flow cytometric analysis of CD4^+^/CD8^+^ T cell ratios in blood, thymus and spleen of 10 week old MMTV-Cre/*Ctsl^−/−^* and LysM-Cre/*Ctsl^−/−^* female mice (Figure S1D,E). The CD4^+^/CD8^+^ cell ratios of those mice were unchanged when compared to the *Ctsl^fl/fl^* control mice, whereas mice with constitutive Ctsl deficiency showed the expected decline in CD4^+^ T cells. This result allowed the further analysis of Ctsl in breast cancer without the interference of a T cell imbalance.

Subsequently, we tested whether Ctsl deletion would affect the differentiation of bone marrow-derived macrophages (Figure S3). During differentiation, *Ctsl^fl/fl^* myeloid cells displayed increasing Ctsl expression, which was not found in LysM-Cre/*Ctsl^−/−^* macrophages, further proving an effective gene targeting (Figure S3A). In vitro differentiated LysM-Cre/*Ctsl^−/−^* macrophages were morphologically similar to wild-type (WT) macrophages (Figure S3B). Analysis of the macrophage markers CD11b^+^/F4/80^+^ and CD11b^+^/CD206^+^ by flow cytometry during differentiation induced by colony stimulating factor 1 (CSF-1) showed homologous marker expression between WT and LysM-Cre/*Ctsl^−/−^* macrophages (Figure S3C). In summary, Ctsl deficiency has no effects either on morphology or on differentiation of bone marrow-derived cells to macrophages.

### 2.2. Ctsl Deletion in Mammary Epithelial Cells Delays Tumor Onset

**Figure 1:**
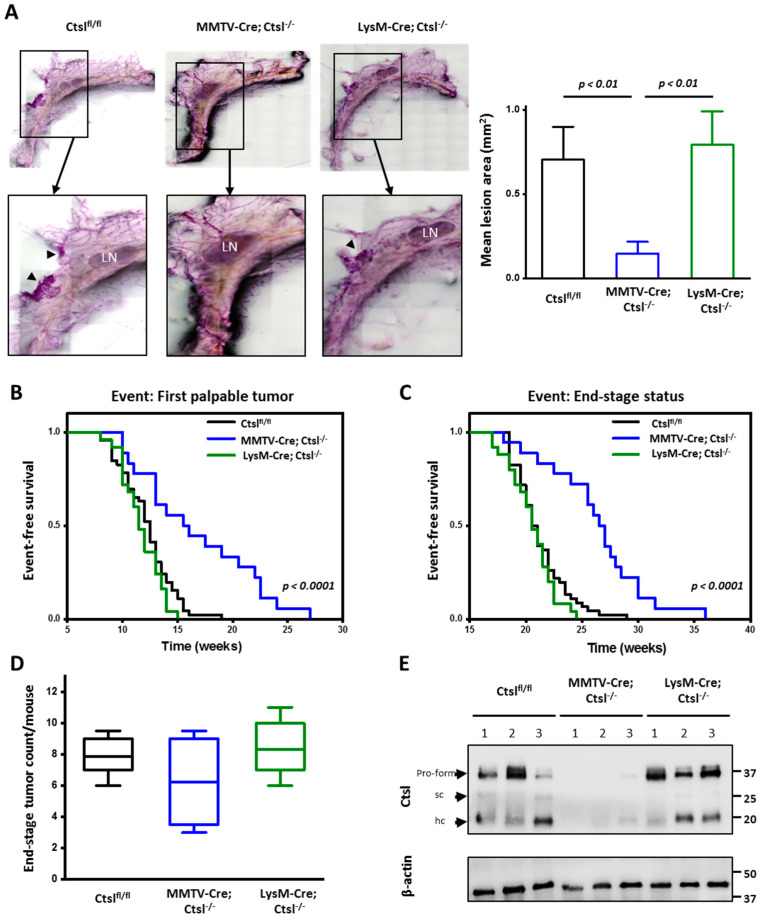
Ctsl deletion in epithelial cells of mammary tumors delay tumor onset. (**A**) Representative images of mammary gland whole mounts of 8-week-old female mice bearing the PyMT^+/T^ antigen and their respective magnification, showing emerging locally-restricted hyperplasia in *Ctsl^fl/fl^* and LysM-Cre/*Ctsl^−/−^* mice. LN, lymph node; arrowheads indicate hyperplasia. Whole mounts were imaged at 4x magnification in an automated manner and stitched together by software; thus, scale bars were not applicable. Quantification of premalignant lesions shows a significant decreased area in MMTV-Cre/*Ctsl^−/−^* mice (*p* < 0.01, Mann–Whitney) (*Ctsl^fl/fl^ n* = 9; MMTVCre/*Ctsl^−/−^ n* = 11; LysM-Cre/*Ctsl^−/−^ n* = 6); mean ± S.E.M. Single images were recorded at 4× magnification, and at least 6x6 frames were stitched together to full images of whole mounts in an automated manner by the Keyence software. (**B**) Tumor-free survival and end-stage determined by palpation of mammary glands starting at 8 weeks of age. (**C**) Significant delay in both tumor onset and end-stage completion was observed upon Ctsl knockout in MMTV-Cre/*Ctsl^−/−^* mice (*p* < 0.0001, Kaplan–Meier) (*Ctsl^fl/fl^ n* = 46; MMTV-Cre/*Ctsl^−/−^ n* = 17; LysM-Cre/*Ctsl^−/−^ n* = 25). (**D**) Count of the multifocal tumors at end-stage was documented prior to sacrifice (*Ctsl^fl/fl^ n* = 46; MMTV-Cre/*Ctsl^−/−^ n* = 17; LysM-Cre/*Ctsl^−/−^ n* = 25). Results were not statistically significant among groups. In the box plots, the boundary of the box closest to zero indicates the 25th percentile, the line within the box marks the median, and the boundary of the box farthest from zero indicates the 75th percentile. Whiskers indicate the 10th and 90th percentiles. (**E**) Immunoblot analysis of Ctsl expression levels in tumor lysates. Expression levels of the three characteristic bands expected for Ctsl (proform at 37kDa; sc, single chain at 25 kDa; hc, heavy chain at 20 kDa) vary among mice and tumor (*Ctsl^fl/fl^ n* = 3; MMTV-Cre/*Ctsl^−/−^ n* = 3; LysM-Cre/*Ctsl^−/−^ n* = 3). Minute amounts of any of the three forms of Ctsl can be detected in the MMTV-Cre/*Ctsl^−/−^* tumors due to the specificity of the Cre-mediated recombination, which enables deletion only in breast epithelial cancer cells, conserving the Ctsl expression in the tumor microenvironment.

For the analysis of Ctsl in breast cancer, MMTV-Cre/*Ctsl^−/−^* and LysM-Cre/*Ctsl^−/−^* mice were further crossed with the transgenic MMTV-PyMT mouse model of metastasizing breast cancer in the C57BL/6 genetic background [29,30]. To determine the effects of Ctsl deletion on the development of pre-malignant adenomas, whole mammary fat pads of eight-week-old female mice bearing the PyMT oncogene were examined (Figure 1A). Quantification of the pre-malignant, carmine red-stained areas showed significantly less adenomas in MMTV-Cre/*Ctsl^−/−^* breasts than in *Ctsl^fl/fl^* or LysM-Cre/*Ctsl^−/−^* glands. All MMTV-PyMT mice developed multifocal tumors, as previously described [29]. Consistent with fewer precursor lesions, first palpable tumors in MMTV-Cre/*Ctsl^−/−^* mice occurred at an median age of 17.5 weeks, which represents a six week delay when compared to *Ctsl^fl/fl^* and LysM-Cre/*Ctsl^−/−^* mice (Figure 1B).

Tumors of *Ctsl^fl/fl^* and LysM-Cre/*Ctsl^−/−^* mice reached end-stage size at week 21.5, while MMTV-Cre/*Ctsl^−/−^* cancers reached end-stage size after week 27.5 (Figure 1C). MMTV-Cre/*Ctsl^−/−^* mice showed a reduced tumor incidence per mouse, supporting our findings of impaired tumorigenesis upon specific deletion of Ctsl in mammary epithelium (Figure 1D). As expected, tumor lysates show the presence of Ctsl in *Ctsl^fl/fl^* and LysM-Cre/*Ctsl^−/−^* samples, and a marked reduction of the protease in MMTV-Cre/*Ctsl^−/−^* tumor lysates (Figure 1E). The faint Ctsl bands in MMTV-Cre/*Ctsl^−/−^* samples are derived from non-epithelial cells in the mammary tumors, e.g., fibroblasts and immune cells. The strong reduction of Ctsl in those samples also indicates an efficient deletion of Ctsl in the cancer cells excluding a mosaic gene deletion.

### 2.3. Distinct Effects of Cell Specific Ctsl-Deficiency on Histological Appearance and Metastasis of MMTV-PyMT Breast Cancers

**Figure 2:**
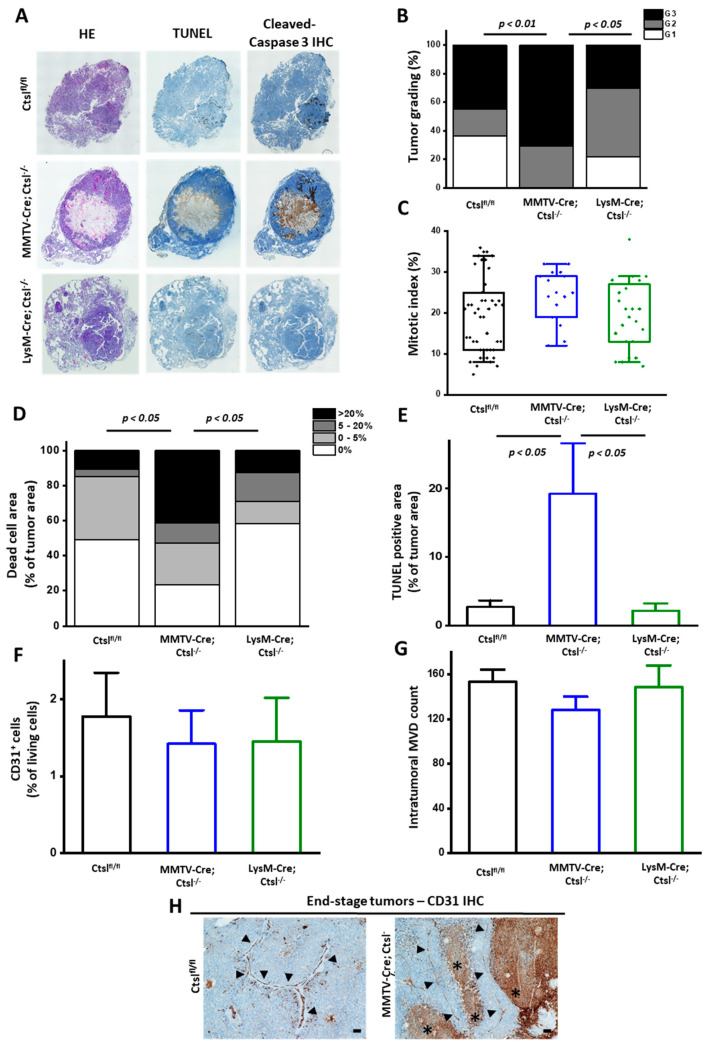
Ctsl knockout in mammary epithelial tumor cells causes tumor dedifferentiation. (**A**) Hematoxylin and eosin (HE) and TdT-mediated dUTP-biotin nick end labeling (TUNEL) staining, as well as cleaved caspase 3 immunohistochemistry (IHC), on end-stage tumors. HE and TUNEL are further quantified in panels B–C of this figure. Caspase 3 IHC is a representative image of 6 tumor specimens analyzed per genotype. Single images were recorded at 4× magnification, and at least 6x6 frames were stitched together to images depicting whole tumors in an automated manner by the Keyence software. (**B**) Blinded histopathological grading of HE stained tumor sections. Grade I (G1), well differentiated carcinoma; grade II (G2), moderately differentiated carcinoma; grade III (G3), poorly differentiated carcinoma (*p* < 0.05; Fischers exact test) (*Ctsl^fl/fl^ n* = 46; MMTV-Cre/*Ctsl^−/−^ n* = 17; LysM-Cre/*Ctsl^−/−^ n* = 25). (**C**) Mitosis count in primary tumor sections (*Ctsl^fl/fl^ n* = 46; MMTV-Cre/*Ctsl^−/−^ n* = 17; LysM-Cre/*Ctsl^−/−^ n* = 25; no significant difference by one-way ANOVA) (**D**) Quantification of dead cell areas by blinded histopathological analysis of HE staining. Large dead cell areas covering almost 50% of tumor area were observed for MMTV-Cre/*Ctsl^−/−^* mice (*p* < 0.05; Fischer’s exact test; *Ctsl^fl/fl^ n* = 46; MMTV-Cre/*Ctsl^−/−^ n* = 17; LysM-Cre/*Ctsl^−/−^ n* = 25). (**E**) Quantification of TUNEL staining of tumor slides (*p* < 0.05, one-way ANOVA, Tukey post-hoc test); mean ± S.E.M. (Ctsl^fl/fl^ *n*=46; MMTV-Cre/*Ctsl^−/−^ n*=17; LysM-Cre/*Ctsl^−/−^ n*=25). (**F**) Analysis of CD31^+^ cells by flow cytometry in end-stage tumors indicate no differences in endothelial cell percentages across genotypes (*Ctsl^fl/fl^ n* = 8; MMTV-Cre/*Ctsl^−/−^ n* = 6; LysM-Cre/*Ctsl^−/−^ n* = 6); mean ± S.E.M. Results were not significantly different between groups (one-way ANOVA, Tukey post-hoc test). (**G**) Microvessel density count performed by an experienced pathologist unaware of the genotype of the tumor specimen (*Ctsl^fl/fl^ n* = 6; MMTV-Cre*Ctsl^−/−^ n* = 5; LysM-Cre/*Ctsl^−/−^ n* = 6); mean ± S.E.M. Results were not significantly different between groups (one-way ANOVA, Tukey post-hoc test). (**H**) Representative images of CD31 immuno-histochemistry stainings of *Ctsl^fl/fl^* (*n* = 6) and MMTV-Cre/*Ctsl^−/−^* (*n* = 5) end-stage tumors showing blood vessels. Notably, blood vessels in MMTV-Cre/*Ctsl^−/−^* surround necrotic areas, ruling out defective vascularization as a cause for extensive cell death linked to the knockout of Ctsl (Scale bar: 100 µm).

Histopathological grading of breast cancer was applied using the Elston/Ellis scoring system. Elston/Ellis unifies nuclear pleiomorphy, mitotic rate, and tubule formation [31]. Grading hematoxylin and eosin (HE)-stained murine tumor slides with this system revealed that MMTV-Cre/*Ctsl^−/−^* cancers appear highly pleomorphic, bear less tubuli, and display more often a solid structure, with similar mitotic rates to *Ctsl^fl/fl^* tumors (Figure 2A,B). This resulted in the classification of most of the MMTV-Cre/*Ctsl^−/−^* tumors as grade 3, in contrast with a lower grading in *Ctsl^fl/fl^* and LysM-Cre/*Ctsl^−/−^* mammary tumors. Additional examples for histology at higher magnification are presented in Figure S2. Notably, the mitotic index of cancer cells was similar in all three mouse cohorts (Figure 2C).

In order to quantify visible dead cell areas in the center of MMTV-Cre/*Ctsl^−/−^* tumors, histological sections were stained with the TdT-mediated dUTP-biotin nick end labeling (TUNEL) method and cleaved caspase 3 as markers for DNA fragmentation and apoptosis, respectively (Figure 2A). Significantly larger necrotic areas comprising dead cells, together with an increased number of apoptotic cells within the tumor mass were detected in MMTV-Cre/*Ctsl^−/−^* cancers (Figure 2D). These results were confirmed by quantification of TUNEL stained areas, which also yielded an increase of dead cells in the MMTV-Cre/*Ctsl^−/−^* group (Figure 2E).

To address insufficient blood supply as a possible cause of cell death in MMTV-Cre/*Ctsl^−/−^* breast tumors we tested their vascularization by detecting the endothelial marker PECAM1/CD31 by immunohistochemistry (IHC). Vascularization of these tumors was not affected by the Ctsl knockout, because flow cytometry analysis yielded similar percentages of CD31^+^ cells in end-stage tumors of all genotypes (Figure 2F). In addition, microvessel density was evaluated by means of CD31^+^ IHC staining of tumor slides by an experienced pathologist in a blinded manner (Figure 2G). Solid clusters, single cells, and endothelial cell clusters with lumina were quantified in the tumor mass within 10 high power fields/400-fold magnification by light microscopy, as reported by Weidner et al. [32]. The vessels were mainly located in the stroma, and there were no differences in microvessel density (MVD) across tumors. Indeed, IHC detection of CD31^+^ cells in *Ctsl^fl/fl^* and MMTV-Cre/*Ctsl^−/−^* tumor slides revealed normal blood vessels even near dead cell areas, confirming no impairment in vessel formation upon Ctsl deletion (Figure 2H).

**Figure 3:**
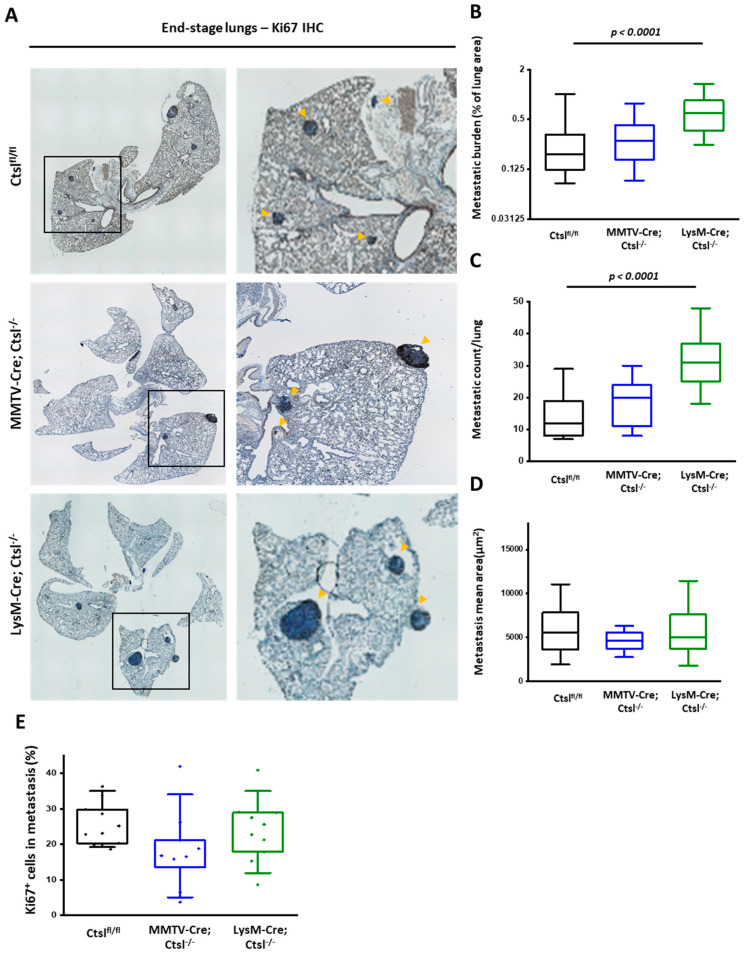
Enhanced lung seeding in end-stage LysM-Cre/*Ctsl^−/−^* tumor mice. (**A**) Representative images of Ki67 IHC stainings of end-stage lungs from *Ctsl^fl/fl^*, MMTV-Cre/*Ctsl^−/−^*, LysM-Cre/*Ctsl^−/−^* conditional knockout mice. Arrowheads highlight the lung metastases, which appear as darker spots within the lung parenchyma. Single images were recorded at 4× magnification and at least 6x6 frames were stitched together to depict whole lungs in an automated manner by the Keyence software. (**B**) Metastatic burden and (**C**) count across all genotypes show a significant increase in metastatic burden and count in the lungs of LysM-Cre/*Ctsl^−/−^* end-stage tumor mice, bearing the deletion in myeloid cells (*p* < 0.0001, Mann–Whitney). Lungs from MMTV-Cre/*Ctsl^−/−^* mice display no differences in their metastatic burden when compared to littermate controls. (**D**) Mean metastasis area remains constant, and thus not significantly changed across genotypes (*Ctsl^fl/fl^ n* = 46; MMTV-Cre/*Ctsl^−/−^ n* = 17; LysM-Cre/*Ctsl^−/−^ n* = 25). (**E**) Ki67-posive proliferating cells in lung metastases (*n* = 7 per group). In the box plots, the boundary of the box closest to zero indicates the 25th percentile, the line within the box marks the median, and the boundary of the box farthest from zero indicates the 75th percentile. Whiskers indicate the 10th and 90th percentiles.

**Figure 4:**
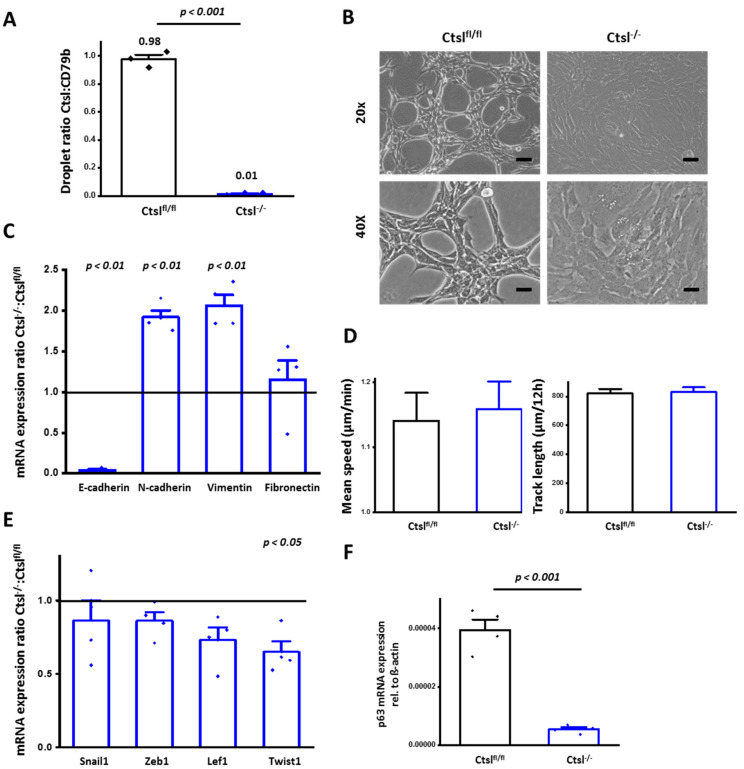
Generation and characterization of *Ctsl^−/−^* cells by in vitro recombination of *Ctsl^fl/fl^* cells. (**A**) Confirmation of Ctsl DNA in three independent batches of *Ctsl^fl/fl^* control cells and in vitro recombined *Ctsl^−/−^* cells by droplet digital PCR quantification. The recipient cells used for in-vitro recombination were a Ctsl floxed mammary epithelial cell line, isolated from an end-stage tumor originating in a female *Ctsl^fl/fl^* (control) mouse. *CD79b* was used as a reference gene, in order to quantify the Ctsl-positive droplets. (*p* < 0.001, Two-tailed *t*-test) (*n* = 4) mean ± S.E.M. (**B**) Bright field microscopy pictures of *Ctsl^fl/fl^* and inducible Cre-transduced mammary epithelial *Ctsl^−/−^* cancer cells in 3-day cultures differ considerably in their morphology and proliferative potential. Cell clusters are observable in *Ctsl^fl/fl^*. Epithelial *Ctsl^−/−^* cancer cells display white dots representing enlarged vesicles (Scale bar: 20×: 50 μm, 40×: 100 μm). (**C**) Quantification of mRNA expression of EMT markers. Significant differences were found in E-cadherin (*p* < 0.01, one-way ANOVA, Tukey post-hoc test), N-cadherin (*p* < 0.001, one-way ANOVA, Tukey post-hoc test), vimentin (*p* < 0.01, one-way ANOVA, Tukey post-hoc test), supporting a phenotype other than epithelial (*n* = 4) mean ± S.E.M. (**D**) Cell tracking assay, monitoring cell movement, mean velocity and path length of cells plated at low density were tracked over 12 hours. Mean velocity and mean track measured by cell tracking showed no difference in the speed and the distance the cells covered during the experiment (*n* = 45); mean ± S.E.M. (**E**) Quantification of mRNA expression of EMT transcription factors Snail1, Zeb1, Lef1, and Twist1. A significant decrease in the transcription levels of Twist1 was found in *Ctsl^−/−^* cells. (*p* < 0.05, one-way ANOVA, Tukey post-hoc test) (*n* = 4), mean ± S.E.M. (**F**) mRNA levels of p63 (*p* < 0.001, two tailed *t*-test) (*n* = 4); mean ± S.E.M.

We further addressed whether metastasis formation could be affected by Ctsl deficiency. Representative pictures of lungs of all three Ctsl genotypes stained for the proliferation marker Ki67 pointed to considerable differences in metastasis (Figure 3A). Strikingly, the metastatic burden was significantly increased in LysM-Cre/*Ctsl^−/−^* mice (Figure 3B). We identified an increased number of metastases as the cause of the increment of the metastatic burden (Figure 3C), whereas the size of the metastases per lung and their Ki67 Proliferation index was not affected (Figure 3D,E). In spite of the undifferentiated high grade cancers of MMTV-Cre/*Ctsl^−/−^* mice, the lung metastatic burden of those animals was identical to the control group. The relevance of pro-inflammatory immune cell infiltration for the migratory and invasive behavior of cancer cells has been widely proven for the PyMT model [33,34,35]. To address the enhanced metastatic burden found in LysM-Cre/*Ctsl^−/−^* mice, we analyzed the numbers of neutrophils, dendritic cells, macrophages, monocytes, T helper cells, cytotoxic T cells, and B cells of end-stage tumors and the corresponding lungs (Figure 4). No significant changes could be found in the percentages of immune cell infiltration either in tumors or in lungs across the three genotypes, pointing towards cell-intrinsic effects of Ctsl in tumor growth and for the development of metastasis.

In summary, Ctsl deficiency in mammary epithelium appears to have opposing phenotypic consequences for the progression of mammary tumors. On the one hand, Ctsl deletion causes pro-malignant loss of differentiation; on the other hand, Ctsl loss induces massive death of tumor cells, which would restrain tumor progression. To address this, we next developed a cell culture model for Ctsl-deletion in MMTV-PyMT breast cancer cells.

### 2.4. Ctsl^−/−^ Mammary Epithelial Cancer Cells De-Differentiate In Vitro

To elucidate cell-specific functions that underlie the previous findings, a Ctsl-deficient cell line was generated by transducing *Ctsl^fl/fl^* breast tumor cells with a doxycycline-inducible Cre-recombinase construct, causing the subsequent recombination of the targeted gene locus. The efficient and homogenous genetic deletion was corroborated by droplet digital PCR (Figure 4A). After three days of culture, *Ctsl^−/−^* breast epithelial cells showed an elongated mesenchymal phenotype, contrasting with the epithelial clusters formed by the parental control cells (Figure 4B). Thus, we analyzed the mRNA expression levels of the epithelial marker E-cadherin, as well as fibronectin, N-cadherin, and vimentin as mesenchymal markers (Figure 4C).

We found that E-cadherin was transcriptionally downregulated, whereas mRNA of N-cadherin and vimentin was increased in *Ctsl^−/−^* cells, with no changes in fibronectin, supporting the previous observation of de-differentiation in MMTV-Cre/*Ctsl^−/−^* cancers. However, in terms of motility, *Ctsl^fl/fl^* and *Ctsl^−/−^* cells showed comparable speed and also analogous track length per time period (Figure 4D). Furthermore, the expression of key transcriptional drivers of epithelial-to-mesenchymal transition (EMT), such as Snail1, Zeb1, and Lef1 were not altered in *Ctsl^−/−^* cells, whereas a decrease in the transcription of Twist1 could be found (Figure 4E). Therefore, the morphological changes and the increase in mesenchymal markers at the transcriptional level in cultured *Ctsl^−/−^* cells is consistent with the de-differentiation observed in vivo for MMTV-Cre/*Ctsl^−/−^* tumors. However, the lack of enhanced motility and the absence of induced EMT-transcription factors largely exclude a canonical EMT process as the cause of the morphological changes in *Ctsl^−/−^* breast tumor cells. In this context, we addressed the transcription levels of p63 (Figure 4F), known for its role in sustaining proliferative potential and stemness of breast epithelial cells [36]. The level of p63 was significantly reduced in four independent batches of *Ctsl^−/−^* cells.

### 2.5. Ctsl^−/−^ Mammary Epithelial Cancer Cells Are Growth Defective 

**Figure 5:**
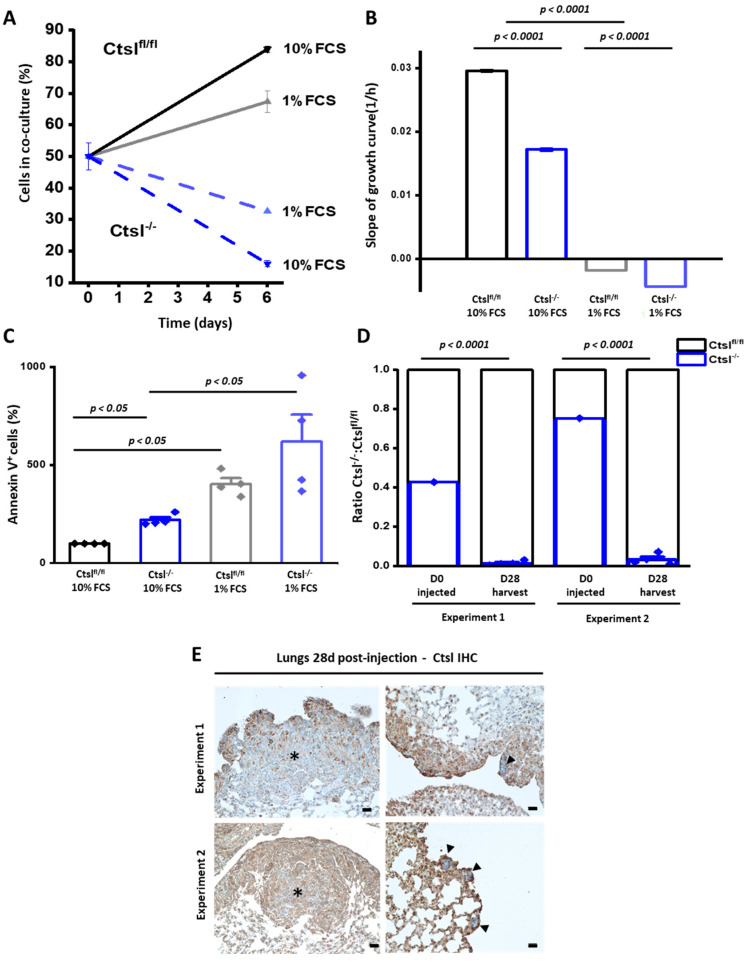
*Ctsl^−/−^* cells display defective proliferation and enhanced cell death. (**A**) *Ctsl^fl/fl^* cells outgrow *Ctsl^−/−^* cells after 6 days of mixed culturing. DNA copy numbers of *Ctsl* and *CD79b*, used as housekeeping genes, were quantified by droplet digital PCR. (**B**) Real-time proliferation analysis by measuring the impedance changes with an x-CELLigence system during 72 h. Negative slopes correlate to detachment of the cells from the plate (*n* = 7) (*p* < 0.0001, one-way ANOVA, Tukey post-hoc test); mean ± S.E.M. (**C**) Annexin V^+^ cells analyzed by flow cytometry at standard culture conditions (10% fetal calf serum (FCS)) and serum reduction (1% FCS) (one-way ANOVA, Tukey post-hoc test; *n* = 4 independent experiments); mean ± S.E.M. (**D**) Experimental metastasis assay using two mice groups injected with *Ctsl^fl/fl^* and *Ctsl^−/−^* cell-co-cultures show a significant difference in the capability of both cells in establishing metastasis in lungs. A first mixture containing around 45% (42.6%) *Ctsl^−/−^* and 55% *Ctsl^fl/fl^* cells was injected into immunodeficient mice by tail vein injection (Experiment 1). Quantification was performed after 28 days post-injection by droplet digital PCR in mashed lung tissue by analyzing the ratio of Cre recombinase and PyMT to distinguish tumor cells. Minute amounts of Cre genomic DNA, representing *Ctsl^−/−^* cells, could be found in the lungs of the injected mice (0.0136%). Similar observations (0.032% of *Ctsl^−/−^* cells) were achieved by repeating the procedure with a higher concentration of *Ctsl^−/−^* cells (75% to 25% *Ctsl^fl/fl^*) (Experiment 2) (*n* = 4 per experiment) (*p* < 0.0001, one sample *t*-test); mean ± S.E.M. (**E**) Exemplary images of IHC for Ctsl in experimental metastasis assay lung slides. Both experiments resulted in large metastasis composed mainly of *Ctsl^fl/fl^* cells, marked with a star (*). *Ctsl^−/−^* cells form isolated micro metastasis, as indicated by arrowheads (Scale bar: 100 µm).

The delayed development of MMTV-Cre/*Ctsl^−/−^* breast tumors, together with the decrease in transcription of p63 in *Ctsl^−/−^* breast epithelial cancer cells lead to a detailed characterization of cell proliferation in vitro. In initial co-culture experiments starting with a 1:1 ratio of *Ctsl^fl/fl^* and *Ctsl^−/−^* cells, quantification of the non-recombined *Ctsl* locus by droplet digital PCR showed that *Ctsl^fl/fl^* cells clearly outgrew *Ctsl^−/−^* cells after six days (Figure 5A). Subsequently, differences in growth of both cell lines were verified by real-time proliferation monitoring, which revealed that *Ctsl^−/−^* cells multiplied significantly less than *Ctsl^fl/fl^*, regardless of serum concentration, i.e., 10% or 1% fetal calf serum (FCS) (Figure 5B). In these experiments, the negative growth slopes in 1% FCS conditions suggest detachment of eventually dying cells from the plates. Therefore the marked impairment in growth of *Ctsl^−/−^* breast cancer cells appeared to be in large part caused by an increase in cell death, as we observed for tumors in vivo (Figure 2). Indeed, the quantification of annexin V binding to apoptotic cells revealed an at least a two-fold increase in apoptosis in *Ctsl^−/−^* cells after three days of culture, which was further propagated upon FCS deprivation (Figure 5C).

In spite of growth impairments of Ctsl^−/−^ breast cancer cells, they were able to establish metastasis in vivo. Thus, we investigated their migratory capacity in vitro, by means of competitive lung colonization experiments (Figure 5D). We injected 40% or 80% *Ctsl^−/−^* tumor cells into the tail-vein of four WT mice per experiment in two independent experiments. The lungs of the animals were harvested on day 28 post-injection. We quantified the occurrence of the PyMT oncogene and the recombination status of the Ctsl locus of PyMT^+/T^ cells by droplet digital PCR on genomic DNA. In both settings the vast majority of PyMT^+/T^ cells in the lung proved to contain an intact homozygous Ctsl gene, thereby further substantiating the growth advantage of *Ctsl^fl/fl^* cells over the *Ctsl^−/−^* PyMT^+/T^ cells.

Next, IHC for detection of Ctsl was performed on lung slides of these mice (Figure 5D). Large macrometastases were positive for Ctsl, whereas micrometastases were devoid of Ctsl staining. We conclude that *Ctsl^−/−^* as well as *Ctsl^fl/fl^* cells are able to disseminate into the lungs and to establish metastatic microlesions. Importantly, only *Ctsl^fl/fl^* cancer cells were able to colonize the lungs during the timespan of the experiment. For interpretation of these results it has to be noted that *Ctsl^fl/fl^* and MMTV-Cre/*Ctsl^−/−^* mice in the primary MMTV-PyMT model had comparable metastatic burdens (Figure 3B). This must be interpreted in the context of Figure 3, presenting results for mice with end-stage tumors, which occur in *Ctsl^−/−^* mice at a higher age (Figure 1C). Hence, *Ctsl^−/−^* breast cancer cells were shown to be able to colonize the lungs only with a long timespan ahead. Comparisons in matched time settings (as in Figure 5D,E), however, underline the compromised in vivo growth of *Ctsl^−/−^* mammary cancer cells.

### 2.6. Expansion of the Lysosomal Compartment of Ctsl^−/−^ Mammary Epithelial Cancer Cells 

**Figure 6:**
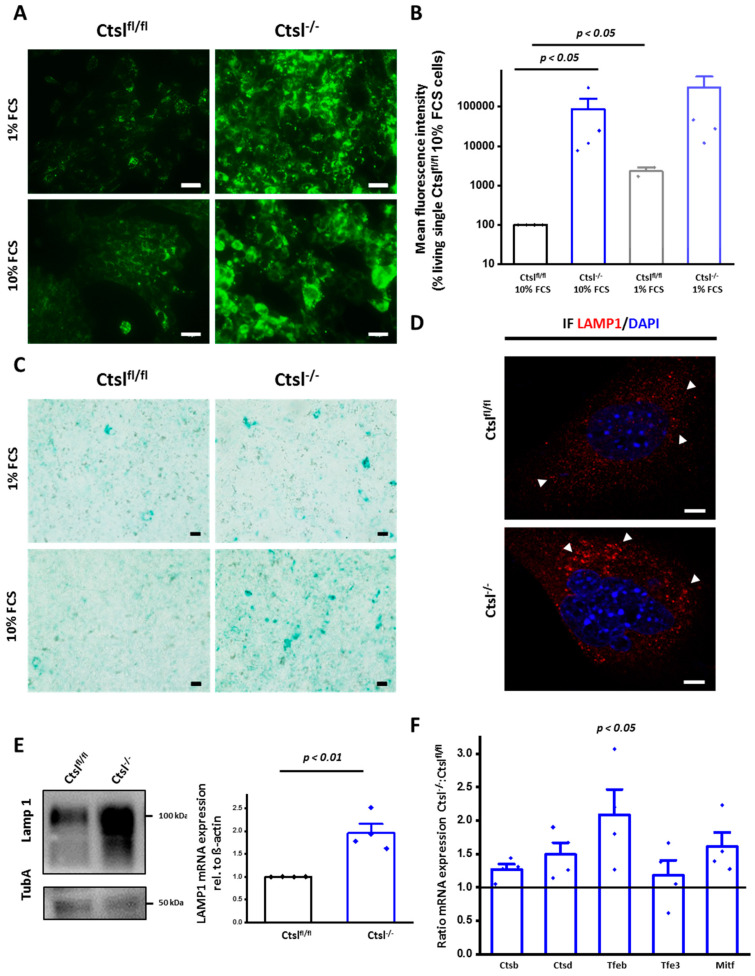
Enlargement and accumulation of acidic vesicles in Ctsl-deficient cells. (**A**) Fluorescence microscopy images of *Ctsl^fl/fl^* and *Ctsl^−/−^* mammary epithelial cancer cells in 3-day cultures under 10% and 1% FCS stained with LysoTracker^TM^ reveal the enlargement of the lysosomal compartment (Scale bar: 50 μm). (**B**) LysoTracker^TM^ mean fluorescence quantification by flow cytometry at standard culture conditions (10% FCS) show a significant increase in green fluorescence consistent with microscopy images. LysoTracker^TM^ positive staining increases under nutrient deprivation conditions (1% FCS) (*n* = 4) (*p* < 0.01, ANOVA, Tukey post-hoc test); mean ± S.E.M. (**C**) Bright field microscopy images of *Ctsl^fl/fl^* and *Ctsl^−/−^* mammary epithelial cancer cells in 3-day cultures under 10% and 1% FCS stained with β Galactosidase (Scale bar: 100 μm). (**D**) Confocal microscopy images of LAMP1 stained as a marker for lysosomes. DAPI was used as nuclear counterstaining (Scale bar: 5 μm). (**E**) Western blot identification of LAMP1 showing its enrichment in *Ctsl^−/−^* cells. Tubulin A was used as a loading control. Quantification of mRNA expression of LAMP1 relative to mRNA expression levels in *Ctsl^fl/fl^* and *Ctsl^−/−^* epithelial breast cancer cells. (**F**) mRNA expression quantification of lysosome-related genes, showing increased transcription of the proteases Ctsb and Ctsd, together with an increase of transcripts of the MiT/Tfe transcription factor family, triggers of lysosomal biogenesis, (one-way ANOVA, Tukey post-hoc test) (*n* = 4); mean ± S.E.M.

Enlargement and accumulation of lysosomes accompanied by defects in the termination of autophagy have been previously observed for *Ctsl^−/−^* cancerous and noncancerous cells [21,23]. In line with this, labeling of *Ctsl^fl/fl^* and *Ctsl^−/−^* breast cancer cells by the acidophilic dye Lysotracker^TM^ revealed a marked accumulation of acidic organelles in Ctsl-deficient cells (Figure 6A). The microscopic aspect was validated by flow cytometry showing a 1000-fold increase of Lysotracker^TM^ mean fluorescence intensity of *Ctsl^−/−^* cells as compared to *Ctsl^fl/fl^* (Figure 6B). Interestingly, *Ctsl^fl/fl^* cancer cells could expand their acidic compartment upon FCS starvation, whereas *Ctsl^−/−^* cancer cells had only a minor increase in staining in this condition. In line with this finding, *Ctsl^−/−^* cells showed an accumulation of lysosomal acidic β-galactosidase activity (Figure 6C), as well as increased occurrence of vesicles positive for the lysosomal membrane protein Lamp1 (Figure 6D). The increase in lysosomal Lamp1 in *Ctsl^−/−^* was further supported by Western blots as well as by mRNA quantification (Figure 6E). In terms of transcription, we also found a trend for increased expression of other lysosomal proteins, such as the proteases cathepsin B and cathepsin D (Figure 6F). Lysosomal biogenesis is mostly triggered by the transcription factors of the MiT/TFE family, especially by the transcription factor EB (TFEB), of which mRNA expression is also augmented in *Ctsl^−/−^* breast cancer cells (Figure 6F). The upregulation of protein transcripts involved in lysosomal biogenesis supports the enlargement of the lysosomal compartment, enabling the cell to maximize its hydrolytic capacity to overcome the accumulation of undegraded cargo and the consequent stress.

### 2.7. Impaired mTORC1 Activity in Ctsl^−/−^ Cancer cells

**Figure 7:**
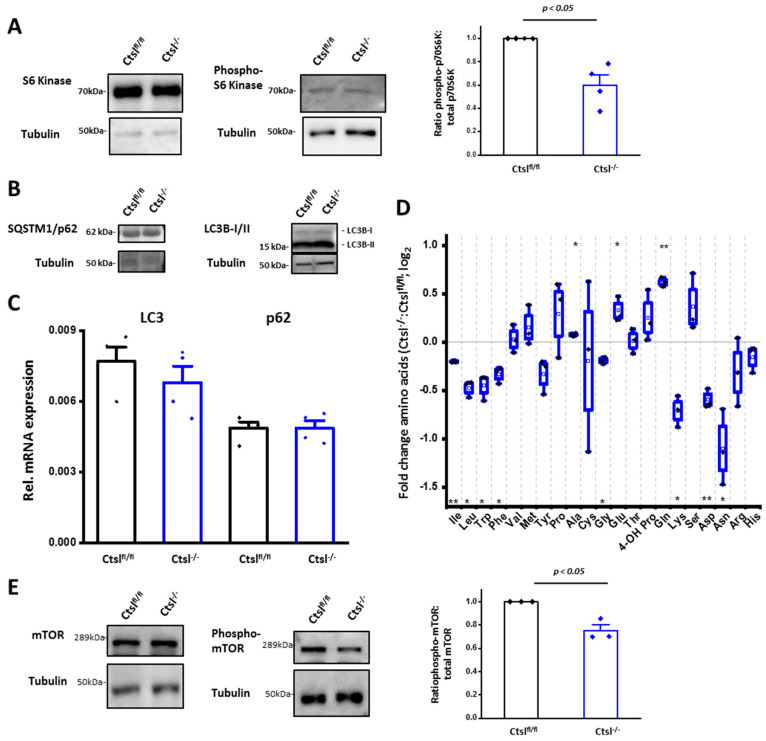
Defective mTOR signaling in *Ctsl^−/−^* cells. (**A**) Representative Western blot images of both isoform p70-S6K and its phosphorylated isoform. Phosphorylation was detected at threonine 389. Western blot quantification of endogenous S6 kinase and phospho-S6 Kinase. The ratios of phosphorylation levels to total proteins are plotted. Phosphorylation of the p70 S6 Kinase isoform is diminished, leading to a significant reduction of the ratio in *Ctsl^−/−^* cells. Alpha-tubulin was used for normalization. (*n* = 4) (*p* < 0.05, one sample *t*-test); mean ± S.E.M. (**B**) Protein expression levels of LC3 I/II and p62 seem to remain unchanged upon Ctsl deletion, resulting in no defects in the autophagic process upon Ctsl deletion. (**C**) Quantification of mRNA expression of the autophagy markers LC3 I/II and p62. mRNA expression levels in *Ctsl^fl/fl^* and *Ctsl^−/−^* cells relative to β-actin for both genes was unaltered, consistent with unchanged protein expression (*n* = 4); mean ± S.E.M. (**D**) Fold change of amino acids in *Ctsl^−/−^* breast cancer cells when compared with *Ctsl^fl/fl^* cells measured by whole cell metabolomics. Alanine, glutamic acid, and glutamine were significantly increased. Significantly reduced levels of isoleucine, leucine, tryptophan, phenylalanine, glycine, lysine, asparagine, and aspartic acid were recorded (*n* = 3, 3 independently generated batches); mean ± S.E.M. (**E**) Representative mTOR and phospho-mTOR Western blot images and its quantification(*n* = 4, *p* < 0.05, one sample *t*-test); mean ± S.E.M. Phospho-mTOR was detected at its serine 2448 residue. Alpha-tubulin was used for normalization.

The mammalian target of rapamycin (mTOR) is a key signaling hub in homeostasis, also involved in the coordination of cellular stress responses [37]. The mTOR complex 1 (mTORC1) requires physical association with adaptor proteins at the cytosolic site of the lysosomal membrane for full activity. mTORC1 inactivation limits cell growth while triggering multiple cellular stress responses, such as lysosomal biogenesis and macroautophagy. Because we had evidence for increased lysosome formation in *Ctsl^−/−^* breast cancer cells (Figure 6), other aspects of mTORC1 signaling were addressed. First, we investigated the phosphorylation state of the p70 S6 Kinase (S6K) isoform as a main mTORC1 downstream target (Figure 7A). We quantified phosphorylation of p70 S6K at threonine 389, as well as the p85 S6K isoform upon phosphorylation at threonine 412. Indeed, quantification revealed a significant decrease in phospho-p70 S6K, indicating reduced mTORC1 activity in *Ctsl^−/−^* cells.

We next focused on the process of macroautophagy. One of its fundamental steps is the conversion of the microtubule-associated protein 1A/1B-light chain 3 I (LC3I) to LC3II. However, the LC3 I/II ratio was similar in *Ctsl^fl/fl^* and *Ctsl^−/−^* cells (Figure 7B). In addition, mRNA expression of LC3 and p62 was unchanged in *Ctsl^−/−^* cells, indicating no differences in the initiation of autophagy (Figure 7C). Our results rule out an increased induction of macroautophagy in *Ctsl^−/−^* breast cancer cells. Moreover, defective terminal degradation is very likely to contribute to the formation of large acidic vesicles in the cells as reported in the previous section.

Therefore, we hypothesized that deficient protein degradation caused by the loss of Ctsl could result in alterations of amino acid levels, which in turn are sensed by mTORC1, modulating its activity [38]. Interestingly, amino acid profiling showed a significant decrease in the ketogenic amino acids, as well as of asparagine and aspartic acid in *Ctsl^−/−^* cells (Figure 7D). In contrast, the remaining glucogenic amino acids were increased or unaffected by the Ctsl-deficiency. This result indicates a considerable metabolic alteration in the *Ctsl^−/−^* breast cancer cells. With regard to the key amino acids critical for mTORC1 activation, leucine concentration was significantly reduced in *Ctsl^−/−^* cells, whereas arginine is also represented at lower levels with a greater variability between experiments. This suggests sensing of those reduced amino acid levels by mTORC1 and hence a lower activity of this complex. To address this, we assessed the status of mTOR phosphorylation at serine 2448 (Figure 7E). Indeed, a significant decrease in mTOR phosphorylation could be established for *Ctsl^−/−^* breast cancer cells.

These results suggest an altered lysosomal turnover as the primary cause of the described cell phenotypes in absence of the lysosomal endoproteinase Ctsl. This affects amino acid availability in cells and mediates a stress response that is, at least in part, triggered by mTORC1 inactivation.

### 2.8. ERK 1/2 Signaling Is Crucial for Viability and Survival of Ctsl^−/−^ Mammary Cancer Cells

**Figure 8:**
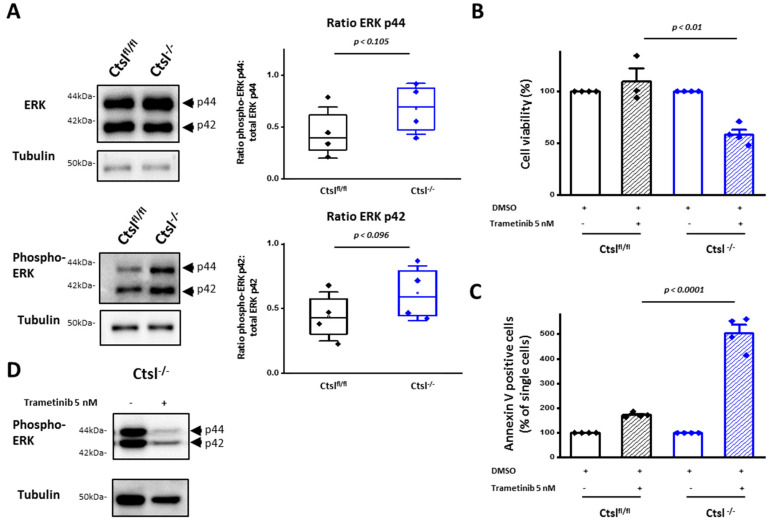
*Ctsl^−/−^* breast cancer cells depend on enhanced phosphorylation of ERK 1 and 2 for survival. (**A**) Representative Western blot images of ERK 1 (p44) and ERK 2 (p42) and respective phospho-ERK 1 and 2, and quantification of the phosphorylation status (*n* = 4; two sample *t*-test); mean ± S.E.M. Phosphorylation was detected at the residues threonine 202 and tyrosine 204 of ERK 1, and the corresponding threonine 185 and tyrosine 187 of ERK 2. Alpha-tubulin was used for normalization. (**B**) Cell viability analyzed upon Trametinib treatment by MTT (*n* = 4, *p* < 0.01, two sample *t*-test); mean ± S.E.M. (**C**) Apoptotic cell death indicated by Annexin V binding upon Trametinib treatment (*n* = 4, *p* < 0.0001, two sample *t*-test); mean ± S.E.M. (**D**) Representative Western blot image corroborating the inhibition of ERK phosphorylation upon Trametinib treatment.

In an attempt to identify intracellular signaling pathways that might help *Ctsl^−/−^* breast cancer cells to cope with the cellular stress resulting from lysosomal dysfunction, we found a trend showing an increase of the phosphorylated isoform of the extra cellular-signal regulated kinases (ERK) 1 and 2 in *Ctsl^−/−^* cells (Figure 8A). In order to study the dependence of our *Ctsl^−/−^* cells on the mitogen-activated protein kinase (MAPK) pathway, cells were treated with Trametinib, a clinically approved inhibitor of the kinase upstream of ERK, i.e., the mitogen-activated protein kinase kinase 1 (MEK) [39]. Cell viability of *Ctsl^−/−^* cells was reduced to 50% after six-day Trametinib treatment (Figure 8B). Apoptotic cell death of *Ctsl^−/−^* cells was massively increased upon treatment, confirming the reliance of the cells on MAPK signaling for survival (Figure 8C). These results point to MAPK signaling as an adaptive cell response to lysosomal stress and suggest a therapeutic benefit for dual targeting of MAPK and lysosomal pathways. A substantial reduction of the phosphorylation of both ERK isoforms, p44 and p42, was corroborated by Western blot upon Trametinib treatment (Figure 8D).

## 3. Discussion

Making use of a newly established mouse line that enables the conditional genetic ablation of Ctsl in the transgenic MMTV-PyMT breast cancer model, we report that Ctsl-deficiency in mammary epithelial tumor cells resulted in a considerable delay in the emergence of tumors. Additionally, end-stage tumors were delayed, morphologically altered and displayed large dead cell areas. In contrast, the selective knockout of Ctsl in myeloid cells had no effects on primary tumors, affecting exclusively the metastatic seeding of PyMT tumor cells to lungs. Hence, our data support a cancer-cell intrinsic role of Ctsl in primary tumor growth while the development of metastasis is influenced by the tumor microenvironment.

A hyperactive PI3K/AKT/mTOR pathway is a characteristic of up to 70% of breast cancers of all molecular breast cancer subtypes, i.e., Luminal A, Luminal B, Her2 (ErbB2)-enriched, as well as basal-like, triple-negative cancers [40]. In MMTV-PyMT mice the expression of the polyoma-virus middle T antigen (PyMT) in mammary epithelium starts in an estrogen-dependent manner in adolescent female mice. PyMT is anchored to the inner leaflet of the plasma membrane, where it becomes swiftly phosphorylated by the SRC proto-oncogene product. The phospho-moieties serve as activating docking sites for intracellular proteins including Shc and PI3K/p85 [41]. Therefore, the MMTV-PyMT mice develop metastasizing breast cancer mainly due to hyperactive mitogen-activated-protein (MAP)-kinase and PI3K signaling pathways, thereby modelling the oncogenic signaling present in a majority of human breast cancers. In addition, breast cancer progression and spontaneous metastasis to the lung of MMTV-PyMT mice strongly depend on pro-tumor polarization of myeloid cells [42]. In summary, the MMTV-PyMT mice are an excellent and widely used model for studying PI3K-related oncogenic signaling and myeloid cell function in breast cancer. Based on transcription profiles, MMTV-PyMT cancers have been related to Luminal-type human breast cancers [43].

The contribution of cysteine cathepsins’ proteolytic activity in the tumor microenvironment has been previously documented [1,44]. Studies transferring PyMT cells in *Ctsb^+/+^* or *Ctsb^−/−^* mice revealed fewer lung colonies, together with an enhanced expression of Ctsb in the lung-infiltrating macrophages, demonstrating a substantial contribution of this protease in promoting lung colonization [6]. In addition, cathepsin S (Ctss) has been shown to be a relevant factor in the promotion of breast-to-brain metastasis, where experimental brain metastasis was only reduced by the combined depletion in macrophages and tumor cells [45]. The deletion of cysteine cathepsins such as Ctss and Ctsz in tumor- associated macrophages (TAMs) contributed not only to reduced metastases but also to limit the invasiveness of lesions in pancreatic cancer [46,47]. However, in contrast to the previous work on Ctsb, Ctss, and Ctsz, in the present study the conditional genetic ablation of Ctsl in myeloid cells increased the metastatic seeding of breast cancer cells to the lungs of the mice. Notably, neither in vitro differentiation of LysM-Cre/*Ctsl^−/−^* bone marrow-derived macrophages (Figure S2), nor the number of immune cells in primary tumors and lungs were significantly altered (Figure S4). Therefore, we hypothesize that Ctsl-deficiency alters polarization and/or function of bone marrow-derived TAMs.

The deletion of the Ctsl in mammary epithelial cells has been shown to have considerable effects on diverse breast cancer traits, exhibiting reduced proliferation, enhanced cell death, and morphological changes. Noteworthy, non-tumor *Ctsl^−/−^* mice raise their litters normally, implying that the lack of Ctsl does not impair the normal structure nor the function of mammary glands [15]. In contrast, we observed a worse histopathological tumor grading in breast cancers lacking Ctsl, with cellular dedifferentiation and loss of normal structural features such as tubuli. We planned to address the mentioned alteration in cell-based approaches, but initially we failed several attempts to establish a *Ctsl^−/−^* cell line by isolating the cells directly from MMTV-PyMT primary cancers and immortalize them in vitro. As presented in Figure 4, we overcame this hurdle by isolating a cell line from a Ctsl-expressing PyMT/*Ctsl^fl/fl^* tumor and subsequently inducing the deletion of the protease by transduction of a Cre recombinase expression system. This allowed to establish a Ctsl-deficient cell model, with following experiments confirming that *Ctsl^−/−^* MMTV-PyMT^+/T^ breast cancer cells display an increased susceptibility for cell death and diminished cell growth. In the search for mechanisms that could explain the observed effects, we first found an increased accumulation of enlarged acidic vesicles. An increase in the amount and volume of vesicles has been already described for many of the spontaneous phenotypes of constitutive *Ctsl^−/−^* mice, in diverse cell types such as keratinocytes [23,24,48], as well as cardiomyocytes [16,49].

The nutrient sensing capability of lysosomes depends on the mTORC1 protein complex [50]. The activation and function of its main effector, the serine/threonine protein kinase mTOR depends on the correct assembly of mTORC1 on the cytosolic side of the lysosomal membrane, where it integrates growth signals and nutrient status such as amino acid levels [37,51]. Importantly, the PyMT oncogene that drives the cancer model used in our study is a strong inducer of the PI3K-AKT-mTOR signaling axis [41].This is relevant because the majority of human breast cancers show a hyper-activation of this pathway due to activating point mutations, gene amplifications, or inactivation of phosphatases such as PTEN [52,53]. In terms of the metabolic state of *Ctsl^−/−^* epithelial breast cancer cells, our data show reduced levels of ketogenic amino acids, suggesting considerable metabolic changes in those cells. Furthermore, the levels of major activators of mTORC1, such as arginine and leucine, are decreased. In line with this, we found evidence of decreased mTOR phosphorylation, as well as of decreased phosphorylation of one of its major targets, the p70 S6 kinase. We propose that deficiency of the lysosomal endoproteinase Ctsl in mammary cancers results in a lysosomal storage phenotype, altered amino acid levels, and interference with mTOR signaling, causing the six-week delay observed for MMTV-Cre/*Ctsl^−/−^* breast tumors. mTORC1 positively regulates cell growth and survival, while negatively regulating lysosomal biogenesis and autophagy [35]. Our in vitro data provided evidence for increased lysosomal biogenesis, rather than macroautophagy induction. Although autophagy can be started by both *Ctsl^fl/fl^* and Ctsl-deficient breast cancer cells, its termination might be impaired in the last cell line. In addition, our findings provide evidence for a growth disadvantage of *Ctsl^−/−^* PyMT^+/T^ cells. This prompted us to search for mechanisms that enable *Ctsl^−/−^* cancer cells to survive and slowly proliferate further. It has been shown for *Ctsl^−/−^* keratinocytes that Ctsl deficiency enhanced recycling of growth factors, such as epidermal growth factor (EGF) [23,54]. PyMT also activates the Raf/ERK cascade to induce tumorigenesis [55]. Thus, we explored the mitogen-activated protein (MAP)-kinase signaling in the *Ctsl^−/−^* PyMT^+/T^ cells. We could find a trend towards increased ERK phosphorylation, which resulted, however, in a significant reduction of cell viability and a major increase in cell death upon MEK inhibition.

## 4. Materials and Methods

### 4.1. Mice

The embryonic stem (ES) cell clone for conditional targeting of mouse Ctsl by a so-called knock-out first strategy (Ctsl^tm1a(EUCOMM)^) was generated by and obtained from the European Conditional Mouse Mutagenesis Program (EUCOMM) (Figure S1A II). The ES cells (JM8.N4; background C57BL/6N) were injected into blastocysts and chimeric mice transmitting the targeted allele were established as founder mice. Subsequently, the neomycine selection cassette and the lacZ reporter were removed by FLPe-mediated recombination of the Frt sites included in the construct (Figure S1A II and III). The resulting C57BL/6 mice harboring the loxP-flanked (floxed) Ctsl gene (*Ctsl^fl/fl^*) were crossed either with the transgenic mouse strains MMTV-Cre^+/T^ or LysM-Cre^+/T^, both bearing the MMTV-PyMT (polyomavirus middle T) antigen. Mice were monitored by palpations twice a week starting at 8 weeks of age until tumors reached end-stage. Animal work was performed in accordance with the German law for animal protection (Tierschutzgesetz) as published on 18 May 18, 2006, with last amendment on 20 November, 2019. Ethics approval registration number is G14/18 RP, regional council Freiburg. Female tumor-bearing mice were monitored by palpations twice a week starting at 8 weeks of age, and tumor onset as well as tumor progression were documented until tumors reached end-stage.

### 4.2. Genotyping

Mouse genotyping was accomplished by PCR. Lysates were diluted and combined with FastGene Taq 2× Ready Mix (#LS31, Nippon Genetics, Europe). PCR reactions were run with a pre-established, optimized protocol for genotyping and end-products were analyzed by gel electrophoresis.

### 4.3. Bone Marrow Isolation and Macrophage Differentiation 

Long bones and hips of 8–10 week-old WT, *Ctsl^fl/fl^* and LysM-Cre/*Ctsl^−/−^* mice were removed and bones were flushed with sterile phosphate buffered saline (PBS). Primary bone marrow cells were incubated with FITC-labeled antibodies (FITC-CD49b #130-116-367, Miltenyi (Bergisch Gladbach, Germany), FITC-CD45R #130-110-708, Miltenyi, FITC-CD3 #130-119-758, Miltenyi, FITC-Ter119 #130-112-719, Miltenyi). Afterwards, cells were incubated with anti-FITC microbeads (#130-048-701, Miltenyi) and sorted with an autoMACS™. The negative fraction containing purified monocytes was further cultured with CSF-1 (#130-101-704, Miltenyi)-supplemented medium and visualized using standard optics (20×/0.35 Ph1) equipped with an AxioCam ERc camera.

### 4.4. Flow Cytometry Analysis of Macrophage Differentiation Stages

To analyze the macrophagic differentiation of monocytes, cells were stained with CD11b (#130-110-554, Miltenyi), F4/80 (#130-116-499, Miltenyi), and CD206 (#141717, Biolegend, San Diego, CA, USA). Finally, cells were washed and fixed in 2% paraformaldehyde. Fluorescence was measured with a MACSQuant^®^ Analyzer.

### 4.5. Mammary Whole Mounts

Mammary fat pads of 8-week-old mice were removed and extended over a glass microscopy slide. Slides were fixed and stained in carmine red-alum solution (2% carmine red, 5% aluminum potassium sulfate). Slides were imaged in an automated manner at 4× magnification and stitched automatically by means of the microscope software package. Slides were stored in methyl salicylate (#M 6752, Merck, Darmstadt, Germany) after imaging [55]. Stained areas were calculated using the Image J software.

### 4.6. Immunohistochemistry

Isolated tumors and lungs were fixed in 4% paraformaldehyde and paraffin-embedded. Five micrometer sections were deparaffinized and stained by different methods. Tumors were stained by HE (hematoxylin-eosin) solution (Merck Millipore, Darmstadt, Germany), or by TUNEL staining using the ApopTag^®^ Peroxidase in Situ Apoptosis Detection Kit, (#S7100, Sigma-Aldrich, Basel, Switzerland), or by Cleaved-caspase 3 (Asp175) IHC (#9664, CST, Danvers, MA, USA). Anti-Ki67 (#ab15580, Abcam, Cambridge, UK) enabled the detection of metastasis in lung slides. Detection was performed using the Vectastain Elite ABC kit (Vectastain ABC HRP kits, #PK-4010 (mouse)/#PK-4001 (rabbit) Vector Laboratories, Burlingame, CA, USA), followed by 3,3-diaminobenzidine (DAB) (Sigma) incubation prior to mounting. Staining of CD 31^+^ cells was performed with APC Rat Anti-Mouse CD31 Clone MEC 13.3 (#551262, BD Biosciences, Allschwil, Switzerland). TUNEL stained areas were quantified using the Image J software.

### 4.7. Histological Grading of Tumors

Histopathological grading and estimation of the necrotic surface were performed on HE-stained tumor sections in a blinded manner by an experienced pathologist using the Elston/Ellis scoring system [31].

### 4.8. Quantification of Metastasis

For the quantification of metastasis, lung slides were stained for Ki67 in order to differentiate rapidly proliferating breast tumor cells from the lung parenchyma. For a correct representation of metastasis across the whole lung, three spatially separated planes per lung were quantified. By means of the free software Image J, the total area of all three lung slides was calculated, together with the areas of the metastasis. The metastatic burden was calculated by dividing the sum of the total area of the metastasis present in each slide by the sum of the total area of the lung, with the total area of the lung set to 100%. The mean size was calculated by dividing the area of the metastasis by the number of metastases in each of the three slides per lung.

### 4.9. Flow Cytometry of Tissues and Cells

For T cell phenotyping, 10-week-old mice were anesthetized and blood was withdrawn. The spleen and thymus were harvested and disrupted by passing through a cell strainer. Cells were seeded at a density of 1 × 10^5^ cells per well and stained with a mixture of PE Rat Anti-Mouse CD8a Clone 53-6.7 (#553033, BD Biosciences) and FITC Rat Anti-Mouse CD4 Clone RM4-5 (#553046, BD Biosciences).

For immune-cell phenotyping, tumors and lungs were dissected carefully from end-stage tumor-bearing mice and disrupted by enzymatic dissociation as performed for tumor cell isolation. Neutrophiles and monocytes were stained with Anti-Mouse Neutrophils (Clone 7/4), (#CL8993F, Cedarlane, Port Clinton, OH, USA), APC Rat Anti-Mouse CD45 Clone 30-F11 (#559864, BD Biosciences), and PE Rat Anti-Mouse Ly-6G and Ly-6C Clone RB6-8C5 (#553128, BD Biosciences). T and B cells were stained with APC Rat Anti-Mouse CD45R/B220 Clone RA3-6B2 (#553092, BD Biosciences), FITC Rat Anti-Mouse CD4, and PE Rat Anti-Mouse CD8a, mentioned before. Macrophages and dendritic cells were stained with FITC Rat Anti-Mouse F4/80 (#MCA497FB, Bio-Rad, Hercules, CA, USA), PE Rat Anti-CD11b Clone M1/70 (#557397, BD Biosciences), and APC Hamster Anti-Mouse CD11c Clone HL3 (#550261, BD Biosciences).

For endothelial cell quantification, tumor cells were stained with APC Rat Anti-Mouse CD31 Clone MEC 13.3 (#551262, BD Biosciences).

For Annexin V and LysoTracker^TM^ analysis, cells were detached after 3-day culture and washed repeatedly in Annexin V binding buffer. Cells were subsequently stained with 1:500 FITC Annexin V (#556419, BD Biosciences) or with 1:5000 LysoTracker™ Green DND-26 (#L7526, Thermo Fisher Scientific, Waltham, MA, USA).

All antibodies/reagents were used in 1:200 dilutions. 7-Aminoactinomycin D (Affymetrix/eBioscience, #00-6993-50) or Propidium iodide (#P4864, Sigma-Aldrich GmbH) were used in all experiments as viability staining in a 1:10,000 dilution. Fluorescence of samples was measured using a CytoFLEX S flow cytometer (Beckman Coulter, Krefeld, Germany) and data were analyzed by the FlowJo software (BD Biosciences).

### 4.10. Microvessel Density (MVD) Measurement

CD31-stained endothelial cells (solid clusters, single cells and endothelial cell clusters with lumina) were quantified within 10 high power fields (HPF)/400-fold magnification by light microscopy by an experienced pathologist in a blinded manner in the tumor mass. The counting was performed according to a modified score reported in Weidner et al. (1991) in tumor hot spots [32].

### 4.11. Generation and Culture of Breast Cancer Primary Cell Lines

End-stage tumors of *Ctsl^fl/fl^* mice were carefully harvested and disrupted by enzymatic dissociation with DNAse I (#DN25, Sigma-Aldrich), Hyaluronidase Type I-S (#H3506, Sigma-Aldrich) and Collagenase Type IV (#C5138, Sigma-Aldrich). Cells were kept as monolayer cultures in a cell culture incubator under sterile conditions at 37 °C, 5% CO_2_, and 91.0% rH. Once spontaneously immortalized after 10–12 passages, mammary breast cancer cells were cultured in Dulbecco’s modified Eagle’s medium (Gibco/Invitrogen, Paisley, UK) supplemented with 1% penicillin/streptomycin, 1% L-glutamine (both from Gibco/Invitrogen), and 10% fetal calf serum (PAN-Biotech, Aidenbach, Germany), in humidified air containing 5% CO_2_. Cells were also kept under low-serum conditions (1%). Cells were transduced with a doxycycline-inducible retroviral Cre-construct (kindly provided by Tilman Brummer). Induction by 2 µg/mL doxycycline took place every 48 h for 6 days. In the indicated experiments, 10 μM of the pan cysteine Cathepsin inhibitor E64d were supplemented to the full medium of *Ctsl^fl/fl^* cells every 48h for 4 days prior to lysis.

### 4.12. RT-Cell Proliferation Monitoring

Cells were seeded at a density of 0.5 × 10^5^ and cultured for 3 days. Proliferation was monitored by means of an xCELLigence DP device (OLS OMNI Life Sciences, Bremen, Germany) for 72 h, using E-16 plates (#2801032, ACEA Biosciences Inc., San Diego, CA USA) following manufacturer’s guidelines.

### 4.13. Droplet Digital PCR, Co-Culture- and Experimental Metastasis Assay

The Ctsl locus was analyzed in 1:10 dilutions of lysates generated from 80% confluent cell culture dishes analyzed by droplet digital PCR. Droplet generation was prepared according to manufacturer’s instructions. *CD79b* was used as house-keeping gene. Primers and probe (all ordered from IDT) for *Ctsl* are as follows: probe: 5′-/56 FAM/TCTCACGCT/ZEN/CAAGGCAATCAGG/3IABkFQ/-3′; forward primer: 5′-CTGAGTGAACAGAACCTTG-3′; reverse primer: 5′-GTCCAGACCTCCATTTTC-3′. Primers and probe for *CD79b* are: probe: 5′-/5HEX/ATTGACCAG/ZEN/ACAGCCACCACCTATGAA/3IABkFQ/-3′; forward primer: 5′-GTCCGAAGAGTCACTATG-3′; reverse primer: 5′-GACCTCCAATTCATGTTTC -3′. PCR amplification took place in a Droplet Digital Thermocycler (Bio-Rad) for PCR amplification and data were analyzed by QuantaSoft software (Bio-Rad).

For *Ctsl^fl/fl^* and *Ctsl^−/−^* co-culture experiments, cells were seeded at a ratio of 1:1 and cultured for a period of 6 days. Lysates were generated and analyzed by droplet digital PCR, as mentioned before.

For experimental metastasis assay, co-cultures containing a different ratio of *Ctsl^fl/fl^* and *Ctsl^−/−^* were harvested at a confluence of 80%. One hundred microliter cell suspension of each of the 2 different cell mixtures containing 42% and 75% *Ctsl^−/−^* cells were injected via tail vein injection in immunodeficient mice separately. After 28 days, mice were sacrificed and lungs were harvested. Right lungs were fixed and embedded in paraffin for histological analysis. Left lungs were lysed overnight and PyMT and Cre DNA were analyzed by droplet digital PCR, performed following the aforementioned standard protocol. Primers and probe for PyMT are as follows: PyMT Probe: 5′-/5HEX/CTA CCA GTC /ZEN/GCC GCC TAA GA/3IABKFQ/-3′; PyMT forward primer: 5′-GGG AAT GGA ATG ATT TCT TC-3‘; PyMT reverse primer 5′-GGCTCC TCA TAA CAG AAT A-3′.

Primers and probe for Cre are following: Cre probe 5′-/56 FAM/TCC CGC AGA /ZEN/ACCTGA AGA TGT T/3IABKFQ/-3′; Cre forward primer: 5′-CAT GGT GCA AGT TGA ATA-3′; Cre reverse primer: 5′-CGC CTG AAG ATA TAG AAG A-3′.

### 4.14. Cell Movement Tracking Assay

Cells were plated at low density, 0.015 × 10^6^ cells per well in 24 well plates until attachment was guaranteed (6 h). Plates were imaged using a JuLI^TM^ Stage real time imaging device for a period of 12 h at standard culture conditions. Cell movement was tracked and mean velocity was calculated using the MTrackJ FIJI/ImageJ plugin.

### 4.15. Immunofluorescence

Cells were grown on coverslips and incubated with primary antibodies recognizing Lamp 1 (# ab24170, Abcam), and phospho–RPS6 (#4858S, CST). Corresponding secondary antibodies anti-rat Alexa 555 and anti-rabbit Alexa 488 were also incubated as the primary antibody. For nuclear counterstaining, cells were stained with 4′,6-Diamidin-2-phenylindol (DAPI) diluted 1:10,000 (#D1306, Thermo Fischer Scientific).

### 4.16. β-Galactosidase Assay and LysoTracker™ Stainings 

*Ctsl^fl/fl^* and *Ctsl^−/−^* cells were grown on coverslips and stained using the Senescence β-Galactosidase Staining Kit (#9860, CST) and LysoTracker™ Green DND-26 (#L7526, Thermo Fisher Scientific) following the manufacturer’s instructions.

### 4.17. Preparation of Protein Lysates 

Cell lysates were prepared on the cell culture plate by adding 0.5 mL of RIPA buffer (50 mM Tris-Cl (pH 8.0), 140 mM NaCl, 1% NP-40, 0.5% sodium deoxycholate, 0.1% SDS) supplemented with protease inhibitors (Complete^®^ inhibitor tablets, Roche, Basel, Switzerland) and phosphatase blockers (PhosSTOP inhibitor tablets, Roche). The cell monolayer was lysed after washing with PBS. Protein concentration was determined by a BCA Protein Assay Kit (Thermo Fisher Scientific, #23225).

### 4.18. SDS-Page and Western Blotting

Thirty to fifty micrograms of lysates were loaded onto 8%, 10%, or 12% SDS-polyacrylamide gels and electrophoretically separated by SDS-PAGE, and transferred to nitrocellulose membranes (Amersham GE Healthcare, Chalfont St Giles, UK) using a wet-blot system (Bio-Rad). They were blocked with 4% BSA in TBS-0.1% Tween and incubated with the primary antibody at a concentration of 1:500 overnight at room temperature. For Western blot analysis of macrophagic differentiation stages, Cathepsin B (#SC-6493, Santa Cruz Biotechnology, Santa Cruz, CA, USA), Cathepsin L (#SC-6501, Santa Cruz Biotechnology), and HSP60 (#12165S, CST) were used as primary antibodies, detected with HRP-conjugated rabbit anti-goat (#P0449, Dako, Jena, Germany), HRP-conjugated rabbit anti-mouse (#P0260, Dako), and HRP-conjugated goat anti-rabbit (#5127, CST).

For general Western blots, primary antibodies used were: Cathepsin L (#AF1515, R&D Systems), Lamp 1 (#3243, CST), LC3B I-II (# 2775, CST), and β-actin (#691001, MP). For mTOR signaling, mTOR (#2972, CST), Phospho-mTOR (#5536, CST), S6 Kinase (#ADI-KAP-CC035-E, Enzo Life Sciences, Farmingdale, NY, USA), Phospho S6 Kinase (#9205, CST), ERK (#4695, CST), Phospho-ERK (#4377, CST), RPS6 (#2217S, CST), phospho–RPS6 (#4858S, CST), and Tubulin (#T9026, Sigma) were used. Membranes were incubated with the corresponding HRP-conjugated secondary antibody (Goat-anti-rabbit, #111-035-003, Jackson ImmunoResearch; Goat-anti-mouse, #A0168, Sigma; rabbit-anti-goat, # A5420, Sigma) for 2 h at room temperature, washed and developed using the Super Signal West Pico Chemiluminescent substrate (#34080, Thermo Scientific), and captured with a Fusion SL Detection System (Vilber Lourmat, Eberhardzell, Germany).

### 4.19. RNA Isolation and RT-PCR

An RNeasy Mini Kit^®^ (#74104, Qiagen) was used to isolate RNA from cell lysates, which was subsequently transcribed to cDNA using the iSCRIPT^®^ cDNA synthesis system (#1708890, Bio-Rad). Primer sequences used for quantitative real-time PCR were as follows: β-actin forward: 5′-ACCCAGGCATTGCTGACAGG-3′; β-actin reverse:5′-GGACAGTGAGGCCAGGATGG-3′; Ctsl forward: 5′-GCACGGCTTTTCCATGGA-3′; Cathepsin L reverse: 5′-CCACCTGCCTGAATTCCTCA-3′. Lamp 1 forward: 5′-GTGACAGGT TTGGGTCTGTGGA-3′; Lamp 1 reverse: 5′-GGTCTGATAGCCGACGTGAC-3′.

To analyze EMT markers following primers were used: E cadherin forward: 5′-GTC TAC CAA AGT GAC GCT GAA G-3′; E cadherin reverse: 5′-GTC TAC CAA AGT GAC GCT GAA G-3′. Fibronectin forward: 5′- GTG GCT GCC TTC AAC TTC TC-3′; fibronectin reverse: 5′- ACG TAC TCC ACA GTG GGT TG -3′. N cadherin forward: 5′-TATATGCCCAAGACAAAGAAACC-3′; N cadherin reverse: 5′-TTGGCAAGTTGTCTAGGGAATAC-3′. Vimentin forward: 5′-TCCCTTGTTGCAGTTTTTCC-3′; vimentin reverse: 5′-GATGAGGAATAGAGGCTGCC-3′. Snail1 forward: 5′-TGG AAA GGC CTT CTC TAG GC-3′; snail1 reverse: 5′-TTC ACA TCC GAG TGG GTT TG-3′. Zeb 1 forward: 5′-TAG CCT TAA GGA AGC AGC CA-3′; zeb1 reverse: 5′-TTA AGG CCA AAG GGA CAC AG-3′. Lef1 forward: 5′-GAA GAT GCT GGA GGA TCG CA-3′; lef1 reverse: 5′-CAA GCG CCG ACT TCC AAA AA-3′. Twist1 forward: 5′-GGA CAA GCT GAG CAA GAT TC-3′; twist1 reverse: 5′-AGA CGG AGA AGG CGT AGC TG-3′. To analyze stemness, the following primers for P63 were used: forward: 5′-CTC TCC ATG CCC TCC AC-3′; reverse: 5′-GAG CAG CCC AAC CTT GCT-3′.

To analyze autophagy markers, the following primers were used: LC3 forward: 2 5′-AGC TTC GCC GAC CGC TGT AA-3′. LC3 reverse: 2 5′-CGG CGC CGG ATG ATC TTG AC-3′. p62 forward: 5′-GTC AGC AAA CCT GAC GGG GC -3′. p62 reverse: 5′-CCG GGG ATC AGC CTC TGT AGA T -3′.

To analyze lysosomal biogenesis, the following primers were used: Cathepsin B forward: 5′- CCT GGG CTG GGG AGT AGA GAA TGG AG -3′. Cathepsin B reverse: 5′- TGG AAA AAG CCC CTA AGG ACT GGA CAA T -3′. Cathepsin D forward: 5′- GTG CAC ATG GAC CAG TTG GA -3′. Cathepsin D reverse: 5′- CAA TAG CCT CAC AGC CTC CCT -3′. RagD forward: 5′-GGC TCC ATC TCA CAG TGA CC-3′. Rag D reverse: 5′-CAT CGT TTG CCC TCT GGT GA-3′. TFEB forward: 5′-CGC CTG GAG ATG ACT AAC AAG-3′. TFEB reverse: 5′-CAC TGG GCA ACT CTT GCT TC-3′. Tfe 3 forward: 5′-AAC AGC AAC GCT CCA AAG AC-3′. Tfe 3 reverse: 5′-CTC GTG GTT AGG GAG AGC AG-3′. Mitf forward: 5′-GGA ACA GCA ACG AGC TAA GG-3′. Mitf reverse: 5′-TGC TTG ATG ATC CGA TTC AC-3′.

### 4.20. Amino Acid Profiling by Metabolomics

Cells were cultured for 72 h under standard conditions (10% FCS) and washed three times with 0.9% NaCl. Monolayer cultures were harvested on ice with 1 mL Metabolomics lysis buffer (90% Methanol, 2.5 μg/mL Isoguanosine hydrate (#NI07234, Carbosynth Limited, Newbury, UK), 1 μg/mL O-Methyl-L-Tyrosine (#H63096, Alfa Aesar/Thermo Fischer Scientific, Schiltigheim, France). Samples were homogenized and transferred to LC-MS glass vials with the injection volume set to 5 μL. A pool of all samples containing 20 μL of each supernatant was used as quality control. Amino acids were separated by a Waters Acquity UPLC BEH Amide column (150 mm × 2.1 mm, 1.8 μm, Waters Corporation, Milford, MA, USA) at 50 °C using a flow rate of 0.6 mL/min with water + 0.1% formic acid as buffer A and acetonitrile (AE70.2, Roth) + 0.1% formic acid as buffer B. Intensities of all 20 amino acids were measured by liquid chromatography-ESI-MS/MS on a 1290 Infinity UHPLC system coupled to a 6460 triple quadrupole mass spectrometer (Agilent Technologies, Santa Clara, CA, USA) via an Agilent Jetstream electrospray ionization source (Agilent Technologies), in a randomized manner. The following gradient was applied: 0.0–0.1 min at 90% B, 0.01–0.02 min to 85% B, 0.02–1 min to 75% B, 1.0–2.0 min to 40% B. Finally, the column was washed for 3 min at 50% B and re-equilibrated for 4 min at 90% B. Total run time was set to 9 min. The applied MS settings were: capillary voltage, 4000 V; nozzle voltage, 500 V; gas temperature, 300 °C; gas flow, 7 L/min; sheath gas temperature, 350 °C; sheath gas flow, 11 L/min; nebulizer pressure, 50 psi. MS/MS spectra were acquired in dynamic multiple reaction monitoring (MRM) mode. Optimized settings and mass spectral transitions were taken from previous reports [56].

### 4.21. MEK Inhibitor (Trametinib) Treatment

*Ctsl^fl/fl^* and *Ctsl^−/−^* cells were seeded separately at a density of 1 × 10^6^ and medium was supplemented with 5 nM Trametinib (GSK1120212), every 48 h for 6 days [39].

### 4.22. MTT Cell Viability Assay

Cells were plated on day 5 of Trametinib treatment at a density of 1 × 10^4^ cells per well in a 96 well transparent plate. After 24 h, 150 μL of a 1:10 dilution of the MTT reagent (#Ab146345, Abcam) in Indicator-free full-DMEM were added. Cells were incubated with MTT reagent until DMSO was added for detection. Absorption was measured at 570 nm and 650 nm with an Enspire Perkin Elmer multimode plate reader.

### 4.23. Statistical Analysis

All quantitative data presented are reported as mean ± standard error of the mean (S.E.M.) of at least 5 biological replicates from independent experiments. Single data points were plotted in the cases where *n* < 5. *n* value, *p* value, as well as statistical test used in each analysis, are stated in the figure legends. One or two sample Student´s *t*-test (two tailed) was used for comparison of the data from experimental groups. Analysis of variance (ANOVA) followed by a post-hoc Tukey test was used for multiple-group comparison. The two-tailed non-parametric Wilcoxon–Mann–Whitney-test was used if data were aberrant from normal distribution. Fischer´s exact test was used to analyze categorical data. Origin 2018^R^ was used to plot the data as well as for its statistical analysis.

## 5. Conclusions

Ctsl deficiency in mammary epithelium strongly impairs lysosomal function, resulting in altered mTOR signaling. To bypass the effects of Ctsl deficiency, *Ctsl^−/−^* cancer cells make use of alternative growth pathways such as the MAP-kinase pathway. Taken together, our findings suggest clinically relevant effects of a combined inhibition of Ctsl and the PI3K/MAP-kinase pathways in breast cancer cells. Furthermore, our study indicates a cancer-cell autonomous role of Ctsl in primary tumor growth, whereas metastasis is also dependent on Ctsl in bone marrow-derived cells. 

## Supplementary Materials

The following materials are available online at https://www.mdpi.com/2072-6694/12/8/2004/s1, Figure S1: Generation of Ctsl conditional knockout mice by means of the Cre-*loxP* technology, Figure S2: Macrophage differentiation remains unaffected upon Ctsl depletion, Figure S3: Features of conditional Ctsl knockout tumors, Figure S4: Unaltered immune infiltration in end-stage tumors and lungs across genotypes. Detailed information about Western blot in Figure 1, Figure 6 and Figure 7.
