# Supplementary material for: Conditional Gene Targeting Reveals Cell Type-Specific Roles of the Lysosomal Protease Cathepsin L in Mammary Tumor Progression
Source: Cancers (Basel). 2020 Jul 22;12(8):2004. doi: 10.3390/cancers12082004 (PMC7463523; doi:10.3390/cancers12082004)
Supplement: Supplementary file 1 [file cancers-12-02004-s001.pdf]

Supplementary Materials

Conditional Gene Targeting Reveals Cell Type-Specific Roles of the Lysosomal Protease Cathepsin L in Mammary Tumor Progression

María Alejandra Parigiani, Anett Ketscher, Sylvia Timme, Peter Bronsert, Manuel Schlimpert, Bernd Kammerer, Arnaud Jacquel, Paul Chaintreuil and Thomas Reinheckel

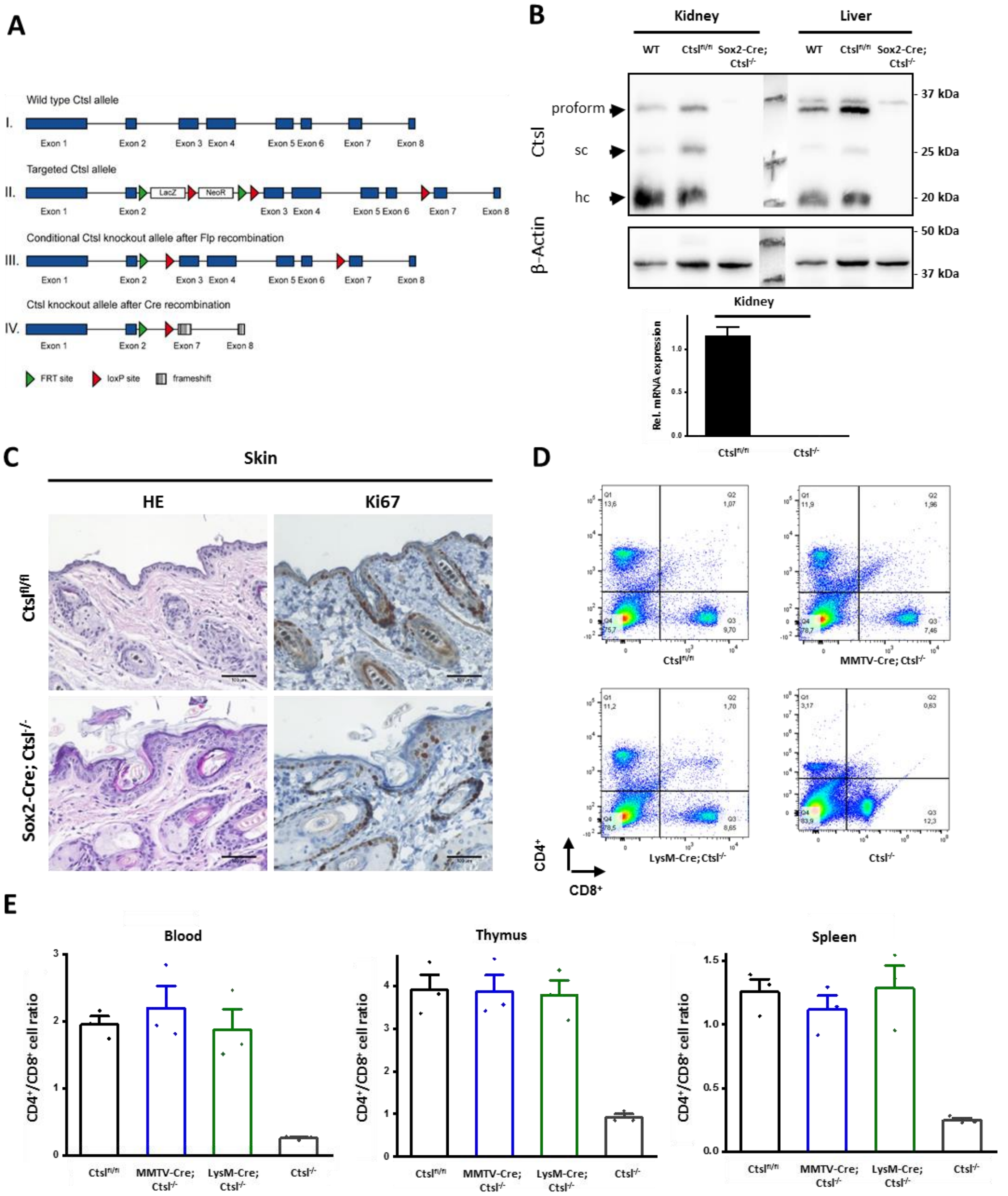

Figure S1. Generation of Ctsl conditional knockout mice by means of the Cre-*loxP* technology. (**A**) Schematic representation of murine wild-type Ctsl gene locus (I). *LoxP* sites introduced in the allele by homologous recombination, resulting in *loxP* sites flanking exons 3–6 (II). Flp recombination to

eliminate cloning-remnant neomycin resistance cassette (III). Subsequent breeding with Cre-transgenic mice drives the recombination of the aforementioned exons, resulting in a tissue-specific conditional Ctsl knockout (IV). (**B**) Western blot analysis of Ctsl protein expression levels in kidney and liver of wild-type (WT), *Ctsl*$^{fl/fl}$, and Sox2-Cre/*Ctsl*$^{-/-}$ mice resembling Ctsl null mice. β-actin was used as loading control. qRT-PCR analysis of mRNA expression levels in kidney relative to β-actin mRNA expression. (**C**) HE and Ki67 immunohistochemistry staining of skin slides showing hyperkeratosis and atypical hair follicles in Sox2-Cre/*Ctsl*$^{-/-}$ mice, resembling the hair phenotype of Ctsl null mice (Scale bar: 100 μm). (**D**) Exemplary dot-plots of spleens of all genotypes where $CD4^+/CD8^+$ cells are analyzed by flow cytometry. (**E**) Comparable $CD4^+/CD8^+$ cell ratios of all genotypes in blood, thymus and spleen analyzed by flow cytometry demonstrate the suitability of our conditional MMTV-Cre/*Ctsl*$^{-/-}$ and LysM-Cre/*Ctsl*$^{-/-}$ knockout models for further cancer studies (*Ctsl*$^{fl/fl}$ $n = 3$; MMTV-Cre/*Ctsl*$^{-/-}$ $n = 3$; LysM-Cre/*Ctsl*$^{-/-}$ $n = 3$; *Ctsl*$^{-/-}$ $n = 3$); mean ± S.E.M. Ctsl null mice were used as a negative control, were the ratio was expected to be significantly smaller due to the aforementioned lack of $CD4^+$ cells.

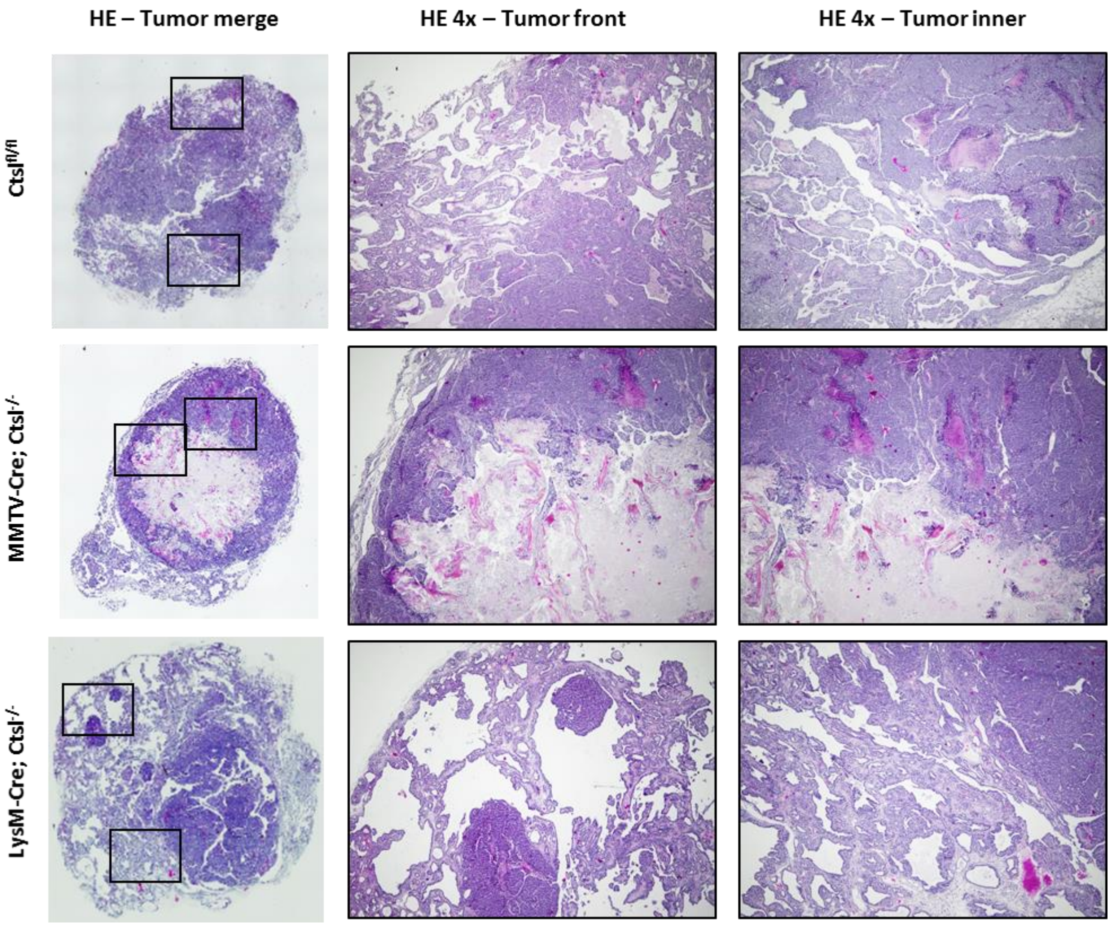

Figure S2. Features of conditional Ctsl knockout tumors. These representative 4× images of all three genotypes display major differences in the tumor structure, supporting the data shown in Figure 2. Major alterations were observed in MMTV-Cre/*Ctsl*$^{-/-}$ tumors regarding tubuli formation and nuclear pleiomorphy, two parameters included in tumor grading, and cell death. Vascularization was not altered across genotypes.

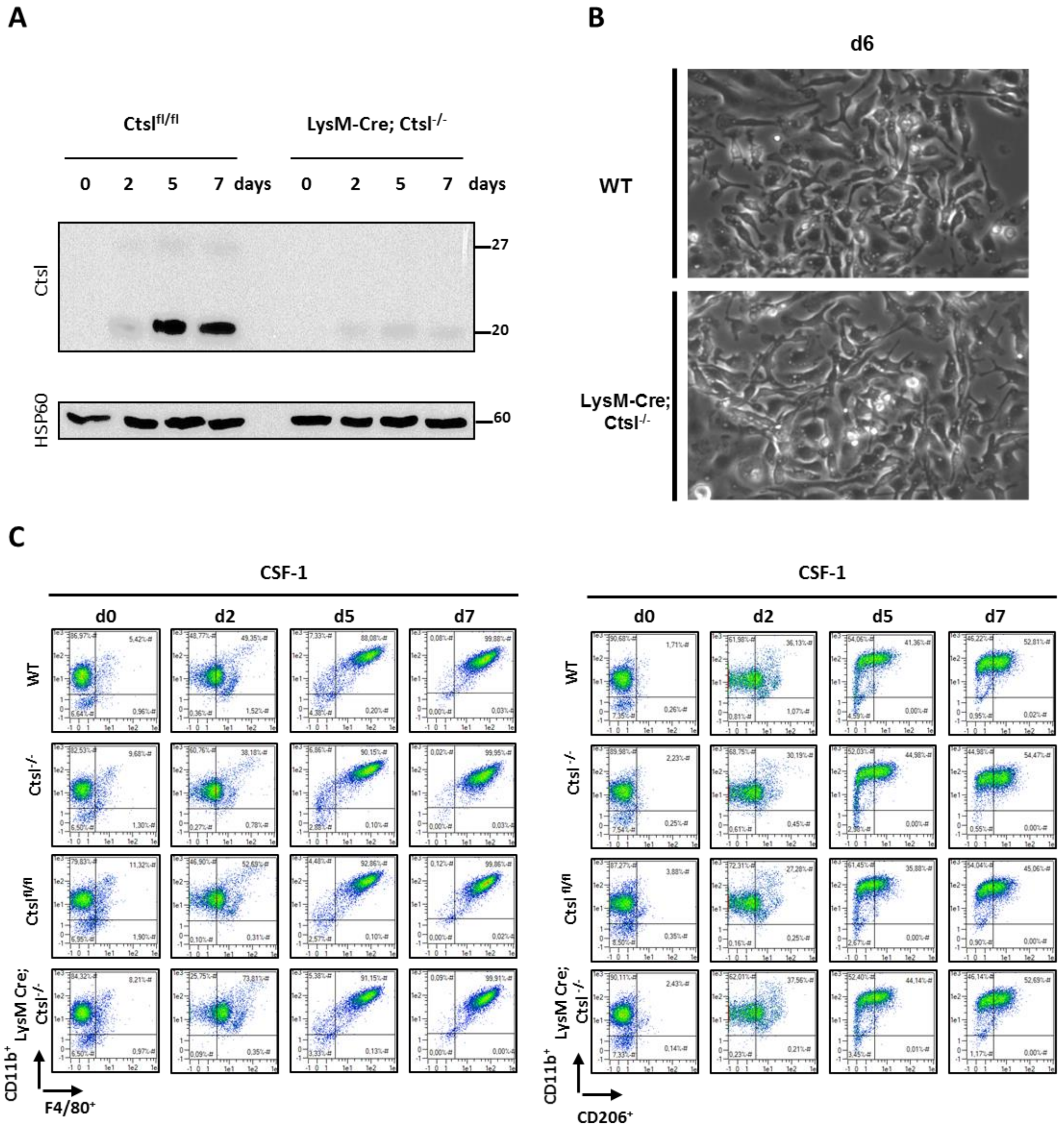

Figure S3. Macrophage differentiation remains unaffected upon Ctsl depletion. (**A**) Immunoblot analysis of Ctsl expression levels of bone marrow isolated cells from *Ctsl*$^{fl/fl}$ and LysM-Cre/*Ctsl*$^{-/-}$ mice after 0, 2, 5, and 7 days of differentiation under M-CSF medium supplementation shows no Ctsl expression in macrophages originating from LysM-Cre mice. (**B**) WT and LysM-Cre/*Ctsl*$^{-/-}$ macrophages after 6 days of differentiation. Knockout macrophages resemble wild type macrophages morphologically. (**C**) Representative dot-plots for wild type, Ctsl null, Ctsl^{fl}/^{fl} and LysM Cre/Ctsl$^{-/-}$ freshly isolated monocytes during differentiation. No differences in macrophage differentiation were observed across WT, Ctsl null, *Ctsl*$^{fl/fl}$ and LysM-Cre/*Ctsl*$^{-/-}$ macrophages upon analysis of typical macrophage markers CD11b^{+}/F4/80^{+} and CD11b^{+}/ CD206^{+} by flow cytometry after 0, 2, 5, and 7 days under CSF-1 treatment.

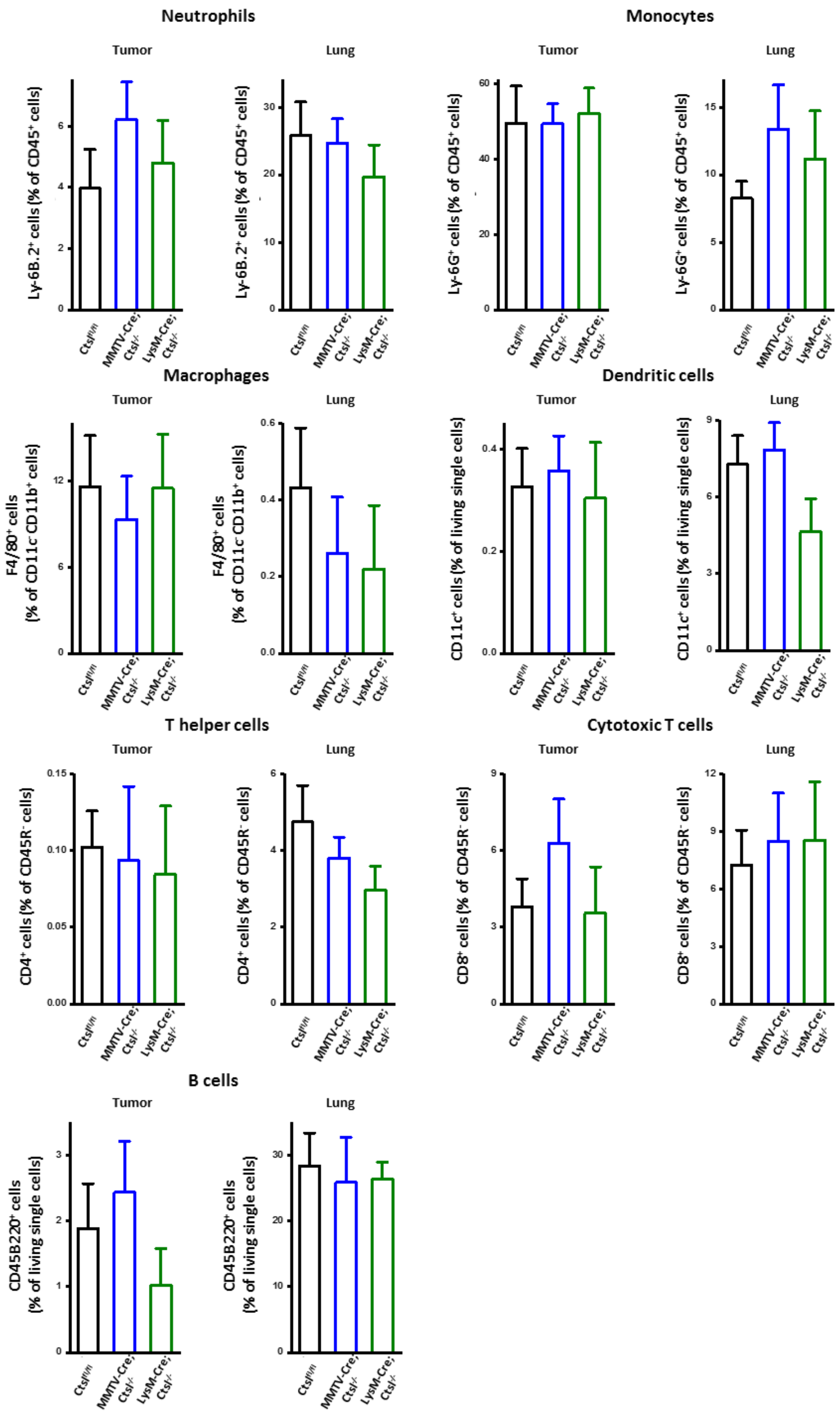

Figure S4. Unaltered immune infiltration in end-stage tumors and lungs across genotypes. Immune cell profiling for neutrophils ($CD45^+$, $Ly6B^+$, $Ly6G^+$), monocytes ($CD45^+$), macrophages ($CD11b^+$, $F4/80^+$), dendritic cells ($CD11c^+$), T helper cells ($CD4^+$), cytotoxic T cells ($CD8^+$), and B cells (CD45R1-$B220^+$) shows no significant alteration in the infiltration percentages of tumors (left) and lungs (right) of end-stage mice ($Ctsl^{fl/fl}$ $n = 8$; MMTV-Cre/$Ctsl^{-/-}$ $n = 6$; LysM-Cre/$Ctsl^{-/-}$ $n = 6$); mean ± S.E.M.

Detailed Information about Western Blot in Figures 1, 6 and 7

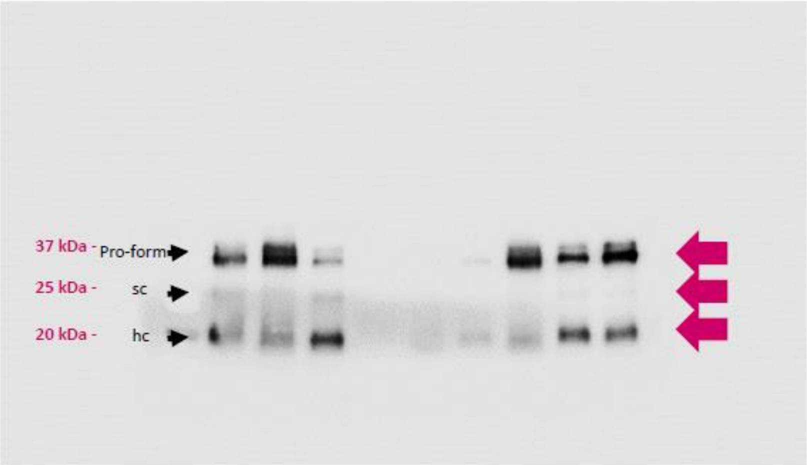

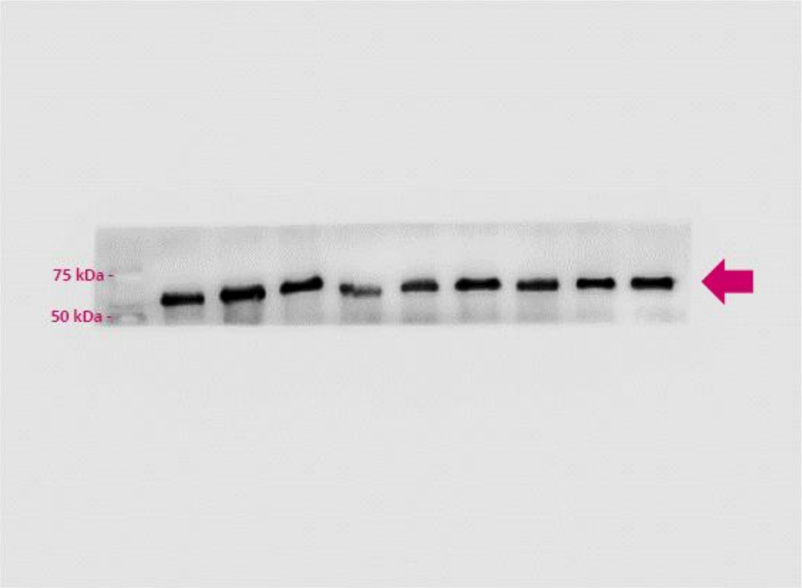

Whole Blots and densitometric analysis of Figure 1 (Figure 1. Ctsl deletion in epithelial cells of mammary tumors delay tumor onset).

Figure 1 Ctsl deletion in epithelial cells of mammary tumors delay tumor onset AUC densitometric reading.

Sample	Lane	Cstl-37 kDa	Lane	Cstl-25 kDa	Lane	Cstl-20 kDa	Lane	Tubulin
Marker	1	Marker	1	Marker	1	Marker	1	Marker
Tumor Lysate Ctsl flox 1	2	6479.426	2	10203.409	2	7101.841	2	7457.134
Tumor Lysate Ctsl flox 2	3	13067.083	3	5630.154	3	6613.134	3	10612.619
Tumor Lysate Ctsl flox 3	4	1853.941	4	5141.790	4	9492.083	4	8455.912
Tumor Lysate MMTV-Cre 1	5	nq	5	nq	5	366.527	5	6225.154
Tumor Lysate MMTV-Cre 2	6	nq	6	nq	6	1065.477	6	6616.426
Tumor Lysate MMTV-Cre 3	7	157.971	7	nq	7	1374.376	7	8395.083
Tumor Lysate LysM-Cre 1	8	11397.134	8	36141.512	8	3592.669	8	7489.669
Tumor Lysate LysM-Cre 2	9	6153.134	9	1140.184	9	6906.962	9	6588.690
Tumor Lysate LysM-Cre 3	10	11024.134	10	1583.891	10	6643.134	10	7960.548

* nq = non quantifiable.

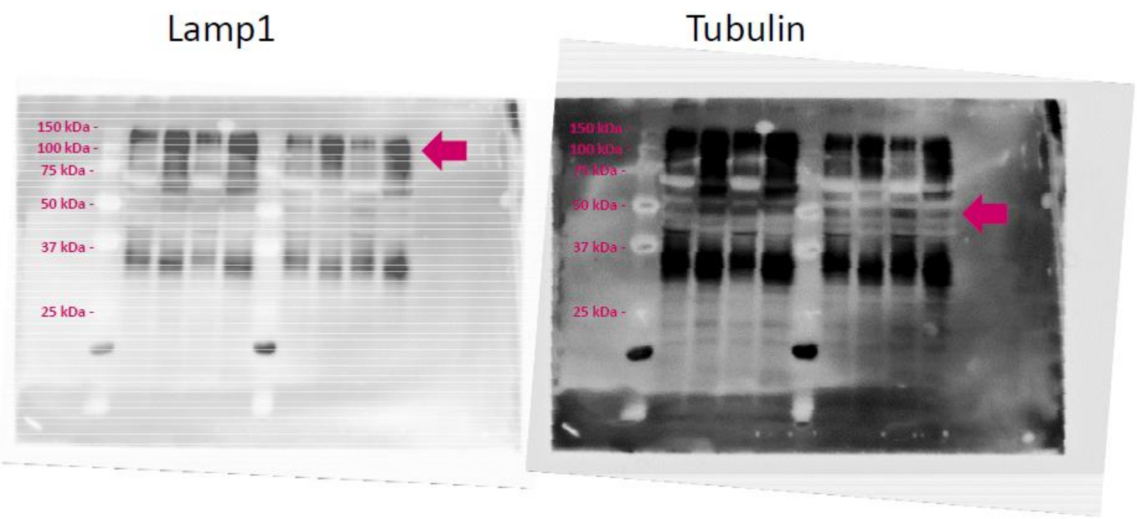

Whole Blots and densitometric analysis of Figure 6 (Figure 6. Enlargement and accumulation of acidic vesicles in Ctsl deficient cells).

Figure 6 Enlargement and accumulation of acidic vesicles in Ctsl deficient cells AUC reading.

Sample	Lane	Lamp1	Lane	Tubulin
Marker	1	Marker	1	Marker
Cstl flox Batch 1	2	7423.52	2	1436.05
Cstl$^{-/-}$ Batch 1	3	10964.45	3	5398.12
Cstl flox Batch 2	4	5131.52	4	735.15
Cstl$^{-/-}$ Batch 2	5	13897.88	5	5251.53
Marker	6	Marker	6	Marker
Cstl flox Batch 3	7	5891.66	7	5586.92
Cstl$^{-/-}$ Batch 3	8	9282.55	8	1860.49
Cstl flox Batch 4	9	3447.01	9	4244.26
Cstl$^{-/-}$ Batch 4	10	11897.03	10	1177.31

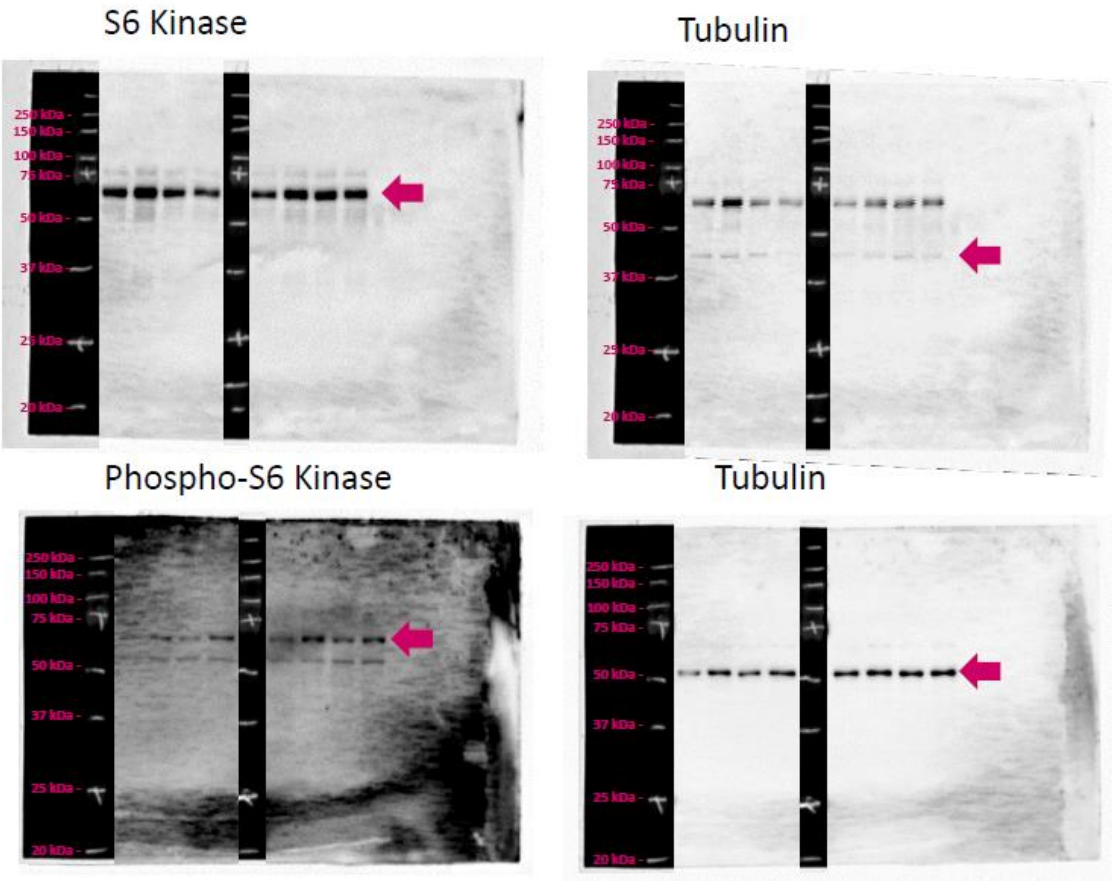

Whole Blots and densitometric analysis of Figure 7, part 1/3 (Figure 7. Defective mTOR signalling is Ctsl $^{-/-}$ cells).

Figure 7 Defective mTOR signalling in Ctsl$^{-/-}$ cells AUC reading (pixel).

Sample	Lane	p70 S6 K	Lane	p70 S6 K Tubulin	Lane	phospho p70 S6 K	Lane	phospho p70 S6 K Tubulin
Marker	1	Marker	1	Marker	1	Marker	1	Marker
Cstl flox Batch 1	2	12192.326	2	9768.309	2	1249.903	2	7192.276
Cstl$^{-/-}$ Batch 1	3	13971.548	3	8212.045	3	9756.170	3	15768.054
Cstl flox Batch 2	4	9189.104	4	6598.045	4	6436.986	4	11567.589
Cstl$^{-/-}$ Batch 2	5	8934.740	5	4526.539	5	9329.321	5	18604.146
Marker	6	Marker	6	Marker	6	Marker	6	Marker
Cstl flox Batch 3	7	9391.447	7	5140.196	7	11018.309	7	18791.589
Cstl$^{-/-}$ Batch 3	8	12189.225	8	5062.447	8	8573.806	8	20586.589
Cstl flox Batch 4	9	10577.255	9	8834.610	9	15690.948	9	19511.640
Cstl$^{-/-}$ Batch 4	10	11023.811	10	5556.832	10	14570.368	10	22809.589

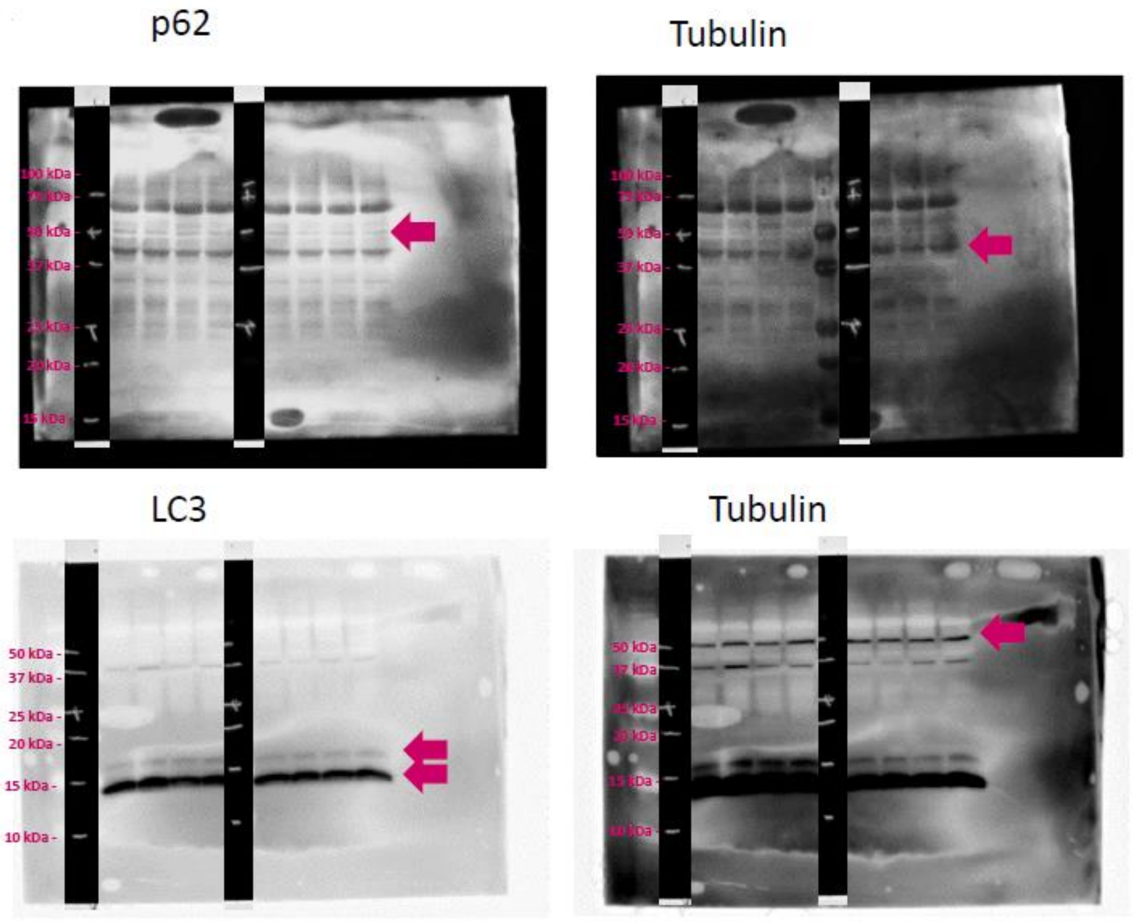

Whole Blots and densitometric analysis of Figure 7, part 2/3 (Figure 7. Defective mTOR signalling is Ctsl$^{-/-}$ cells).

Figure 7 Defective mTOR signalling is Ctsl$^{-/-}$ cells AUC densitometric reading (pixel).

Sample	Lane	LC3 I	Lane	LC3 II	Lane	LC3 tubulin	Lane	p62	Lane	p62 tubulin
Marker	1	Marker	1	Marker	1	Marker	1	Marker	1	Marker
Cstl flox Experiment 1	2	12734.329	2	17144.605	2	4870.095	2	11872.489	2	5963.205
Cstl$^{-/-}$ Experiment 1	3	5409.735	3	5525.773	3	4082.790	3	9341.711	3	3461.560
Cstl flox Experiment 2	4	3991.752	4	1291.518	4	565.790	4	10144.569	4	5616.246
Cstl$^{-/-}$ Experiment 2	5	21921.990	5	16265.726	5	5359.024	5	10829.924	5	1696.447
Marker	6	Marker	6	Marker	6	Marker	6	Marker	6	Marker
Cstl flox Experiment 3	7	14284.161	7	23214.262	7	6038.439	7	12894.468	7	5189.933
Cstl$^{-/-}$ Experiment 3	8	4114.045	8	5026.731	8	306.698	8	10946.640	8	4561.740
Cstl flox Experiment 4	9	3860.551	9	4816.518	9	352.506	9	12256.497	9	5075.882
Cstl$^{-/-}$ Experiment 4	10	7605.120	10	13637.889	10	2660.882	10	11871.681	10	4977.347

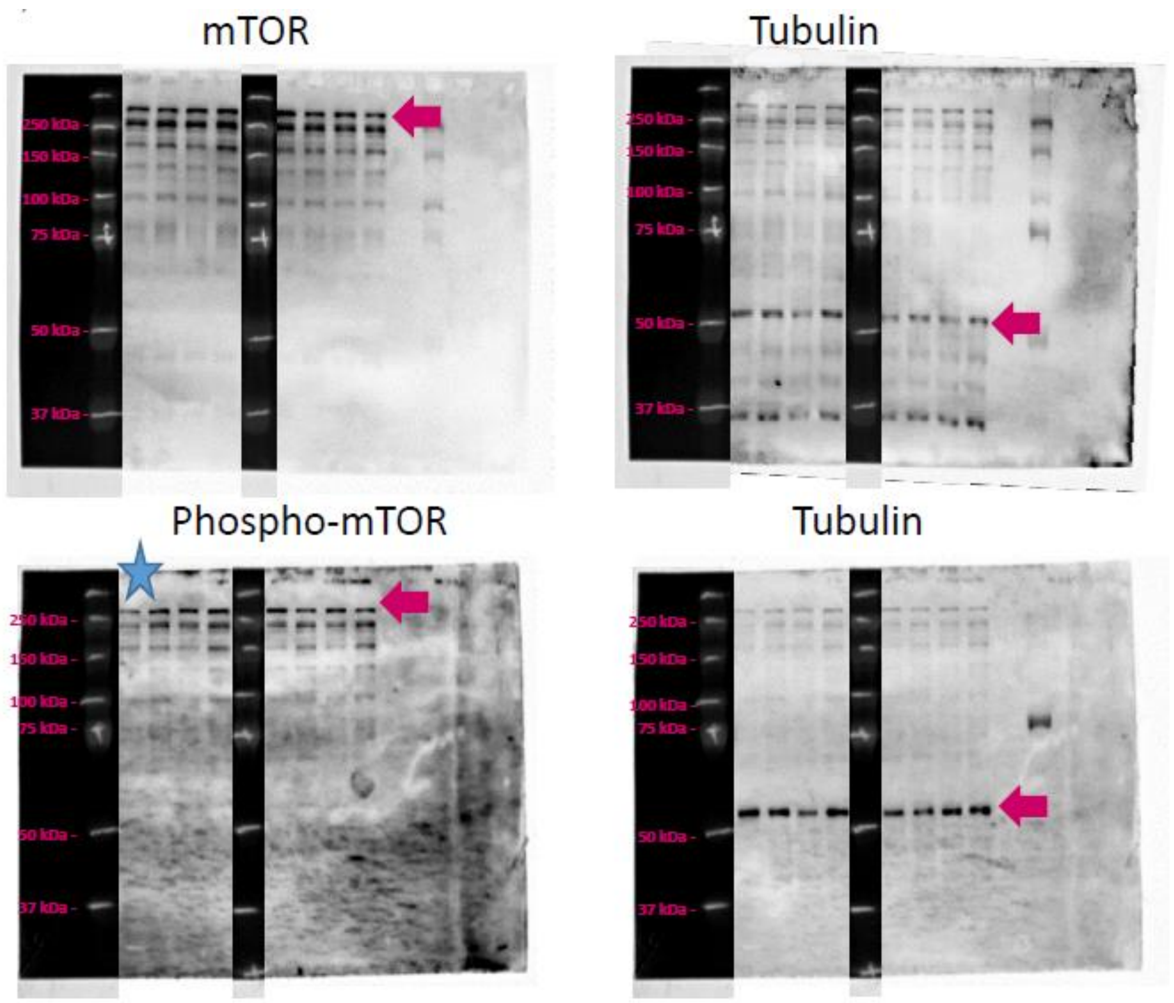

Whole Blots and densitometric analysis of Figure 7, part 3/3 (Figure 7. Defective mTOR signalling is Ctsl-/- cells).

★ Due to transfer problems, Ctsl flox and the corresponding Ctsl-/- sample of Experiment 1 were not taken into account for tha analysis.

Figure 7 Defective mTOR signalling in Ctsl-/- cells AUC densitometric reading (pixel).

Sample	Lane	mTOR	Lane	mTOR Tubulin	Lane	phospho mTOR	Lane	phospho mTOR Tubulin
Marker	1	Marker	1	Marker	1	Marker	1	Marker
Cstl flox Experiment 1	2	12659.569	2	17083.823	2	1429.941	2	14060.983
Cstl-/- Experiment 1	3	12203.296	3	16848.832	3	11648.740	3	10410.376
Cstl flox Experiment 2	4	11998.20	4	11423.76	4	12269.933	4	6745.447
Cstl-/- Experiment 2	5	12892.983	5	18709.924	5	11333.912	5	13513.589
Marker	6	Marker	6	Marker	6	Marker	6	Marker
Cstl flox Experiment 3	7	13831.326	7	11765.167	7	12611.154	7	8513.225
Cstl-/- Experiment 3	8	9686.154	8	11644.246	8	7215.790	8	8044.740
Cstl flox Experiment 4	9	11290.196	9	12408.782	9	10356.690	9	10074.669
Cstl-/- Experiment 4	10	7908.083	10	12544.832	10	6138.731	10	12324.175

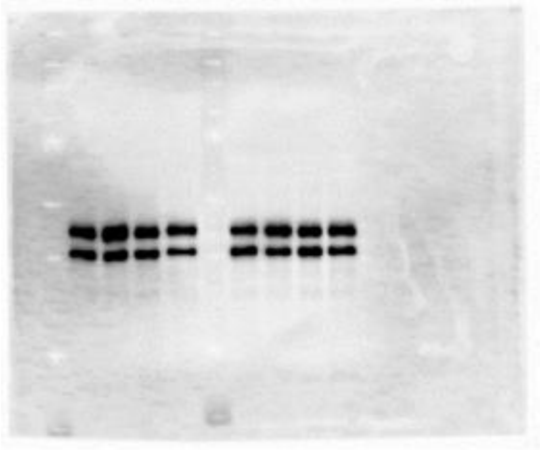

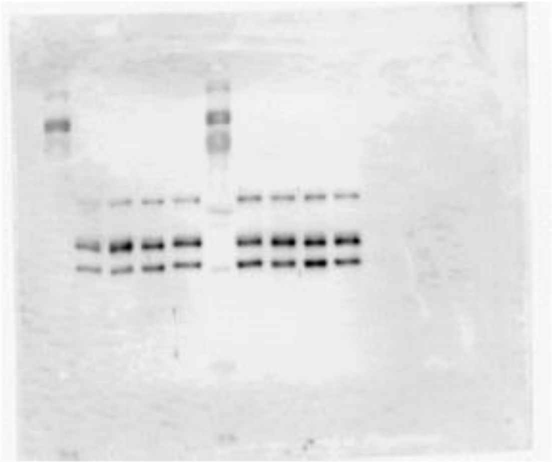

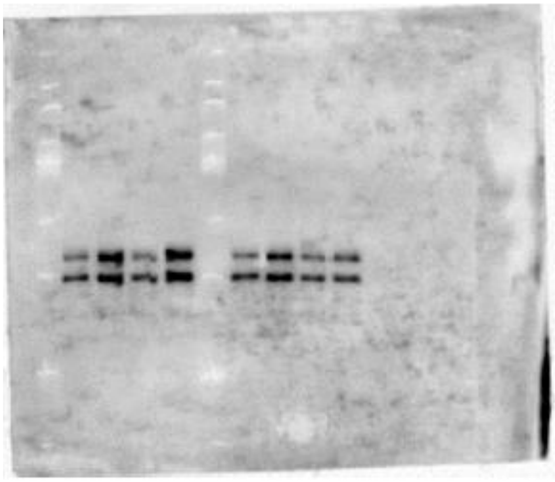

© 2020 by the authors. Licensee MDPI, Basel, Switzerland. This article is an open access article distributed under the terms and conditions of the Creative Commons Attribution (CC BY) license (http://creativecommons.org/licenses/by/4.0/).
